# ALKBH5-HOXA10 loop-mediated JAK2 m6A demethylation and cisplatin resistance in epithelial ovarian cancer

**DOI:** 10.1186/s13046-021-02088-1

**Published:** 2021-09-08

**Authors:** Sipei Nie, Lin Zhang, Jinhui Liu, Yicong Wan, Yi Jiang, Jing Yang, Rui Sun, Xiaolling Ma, Guodong Sun, Huangyang Meng, Mengting Xu, Wenjun Cheng

**Affiliations:** Department of Gynecology, the First Affiliated Hospital of Nanjing Medical University, Nanjing, 210029 Jiangsu China

**Keywords:** N6-methyladenosine, Cisplatin resistance, Epithelial ovarian cancer, ALKBH5

## Abstract

**Background:**

Chemotherapy resistance remains a barrier to improving the prognosis of epithelial ovarian cancer (EOC). ALKBH5 has recently been shown to be one of the RNA N6-methyladenosine (m6A) demethyltransferases associated with various cancers, but its role in cancer therapeutic resistance remains unclear. This study aimed to investigate the role of AlkB homolog 5 (ALKBH5) in cisplatin-resistant EOC.

**Methods:**

Functional assays were performed both in vitro and in vivo. RNA sequencing (RNA-seq), m6A-modified RNA immunoprecipitation sequencing (MeRIP-seq), chromatin immunoprecipitation, RNA immunoprecipitation, and luciferase reporter and actinomycin-D assays were performed to investigate RNA/RNA interaction and m6A modification of the ALKBH5-HOXA10 loop.

**Results:**

ALKBH5 was upregulated in cisplatin-resistant EOC and promoted cancer cell cisplatin resistance both in vivo and in vitro. Notably, HOXA10 formed a loop with ALKBH5 and was found to be the upstream transcription factor of ALKBH5. HOXA10 overexpression also facilitated EOC cell chemoresistance both in vivo and in vitro. Collective results of MeRIP-seq and RNA-seq showed that JAK2 is the m6A-modified gene targeted by ALKBH5. The JAK2/STAT3 signaling pathway was activated by overexpression of the ALKBH5-HOXA10 loop, resulting in EOC chemoresistance. Cell sensitivity to cisplatin was rescued by ALKBH5 and HOXA10 knockdown or inhibition of the JAK2/STAT3 signaling pathway in EOC cells overexpressing ALKBH5-HOXA10.

**Conclusions:**

The ALKBH5-HOXA10 loop jointly activates the JAK2/STAT3 signaling pathway by mediating JAK2 m6A demethylation, promoting EOC resistance to cisplatin. Thus, inhibition of the expression of the ALKBH5-HOXA10 loop may be a potential strategy to overcome cisplatin resistance in EOC.

**Supplementary Information:**

The online version contains supplementary material available at 10.1186/s13046-021-02088-1.

## Background

Epithelial ovarian cancer (EOC) is the most fatal gynecological malignancy worldwide [1]. Platinum-based regimens are currently the first-line chemotherapeutic modality for EOC. However, while it is initially effective, most EOC patients have a poor prognosis owing to the inherently high rate of recurrence and resistance to cisplatin agents [2]. Unfortunately, evidence supporting the appropriate approach against platinum chemoresistance in EOC is limited because of its complicated mechanism. Therefore, the mechanism underlying platinum chemoresistance in EOC needs to be explored to identify potential therapeutic targets to overcome resistance and improve prognosis.

Several mechanisms, including activation of oncogenes, mutation of antioncogene, and dysregulation of cancer-associated signaling pathway, are involved in chemoresistance [3–5]. N6-methyladenosine (m6A) modification is one of the most prevalent mRNA modification and influences mRNA transcription, stabilization, and translation [6]. The core molecules of the m6A methyltransferase complex, including methyltransferase-like 3, methyltransferase-like14, and WT1-associated protein, mediate the methylated modification [7]. Meanwhile, m6A erasers, including fat mass and obesity-associated and AlkB homolog 5 (ALKBH5), act as demethylases to reverse the m6A modifications. The fate of m6A-modified mRNA depends on specific m6A readers that contribute to various processes such as mRNA splicing, degradation, and translation [8].

Previous studies have shown that m6A modifications are involved in important cancer processes, including treatment resistance in hepatocellular carcinoma, breast cancer, and non-small-cell lung cancer [9–11]. Hao et al. recently suggested that ALKBH5 inhibited the progression of bladder cancer and sensitized bladder cancer cells to cisplatin through a casein kinase 2 α-mediated glycolysis pathway [12]. However, research on the role of m6A modification in EOC resistance to cisplatin is limited. ALKBH5 was reported to be overexpressed in EOC tissues and promoted cancer progression by inhibiting EOC cell autophagy [13]. ALKBH5 was also suggested to contribute to resistance to PARP inhibitors in BRCA1/2-mutated ovarian cancer cell lines by regulating the expression of FZD10 mRNA and mediating the Wnt signaling pathway [14]. These data suggest that ALKBH5 plays a significant role in EOC, and it might be a potential therapeutic target.

Thus, this study aimed to explore the role of ALKBH5 and the underlying regulatory mechanism in cisplatin-resistant EOC.

## Materials and methods

### Clinical sample collection

All patients signed informed consent before using clinical specimens. The use of specimens for this study has been proved by the ethics committee of the First Affiliated Hospital with Nanjing Medical University. Tumor tissues were obtained from patients who have undergone surgery in the First Affiliated Hospital with Nanjing Medical University (Jiangsu Province Hospital) between January 2015 to January 2019. Surgically resected specimens were immediately flash-frozen in liquid nitrogen for further investigation. According to National Comprehensive Cancer Network Guidelines, EOC patient who do not achieve complete clinical remission (CR) after initial platinum-based chemotherapy or tumor recurred within 6 months after CR is defined as a platinum-resistant case. Patients with a cisplatin-free interval longer than 6 months are defined as a platinum-sensitive case [15]. In our study, we obtained 15 platinum-sensitive samples and 9 platinum-resistant samples. All 24 patients underwent at least 6 courses of platinum-based adjuvant chemotherapy after surgery. The descriptive information of sensitive- and resistant- patients was provided in Supplementary Table 1. Due to the strict surgical indication of recurrent EOC, the second operation of recurrent cisplatin-resistant EOC is not recommended. In the present study, platinum-resistant samples were obtained from patients who did not achieve CR or tumor recurred within 6 months after the first-line treatment. Meanwhile, different pathological types of EOC samples were also collected to investigate the ALKBH5 and HOXA10 expression (*n* = 57).

### Cell lines and culture conditions

The cell lines HO8910, A2780, A2780-DDP, HO8910-DDP were cultured in RPMI1640 (Gibco) supplemented with 10% FBS (Gibco) and 1% penicillin/streptomycin at 37 °C supplied with 5% CO2. Among these cell lines, HO8910 and A2780 are cisplatin-sensitive EOC cell lines. A2780 and HO8910 underwent continuous stepwise exposure to increasing concentrations of cisplatin to create the cisplatin-resistant cell lines, A2780-DDP and HO8910-DDP.

### RNA extraction and quantitative real-time PCR (qPCR)

According to the manufacturer’s instructions, total RNA was extracted from cultured cells and tissues with Trizol (Invitrogen). Then, cDNA was prepared by HiScript Q RT SuperMix for qPCR (Vazyme). The qPCR was performed with an SYBR Green PCR Kit (Vazyme). The sequences of gene primers used for qPCR were showed in Supplementary Table 2.

### Protein extraction and Western blot assay

Total protein from cultured cells and tissues were lysed in RIPA buffer (Beyotime) with protease inhibitor (Beyotime) and then quantified by using a BCA assay kit (Beyotime). The quantified protein was treated at 100 °C for 5 min and separated by 10% SDS-PAGE buffer. Western blot assays were performed as the protocol as we previously reported [16]. The antibodies used for western blot assay were listed in Supplementary Table 3.

### Lentivirus infection and siRNA transfection

Lentiviral vectors pGC-FU-3FLAG-CBh-gcGFP-IRES-puromycin respectively encoding transcript ALKBH5 (oe-ALKBH5) and HOXA10 (oe-HOXA10) transcripts were purchased from Genechem (Shanghai, China), and cells were incubated with lentivirus and 4 mg/mL polybrene for 24 h. Puromycin (0.5 μg/mL) was added to the medium for selection.

All siRNA and plasmid were transfected in cells by using Lipofectamine 3000 (Invitrogen). Cell assays and RNA extraction were performed after 24 h, and protein extractions were performed after 48 h. All siRNAs were synthesized by GemmaPharma (Shanghai, China), and the RNAi oligonucleotides sequences (5′-3′) were showed in Supplementary Table 4.

### Cell proliferation assays

For Cell Counting Kit-8 (CCK8) assay, EOC cells were plated at a density of 6000 cells per well in 96-well plates. After cell adherence, cell proliferation was determined at 0, 24, 48, and 72 h; CCK-8 (Vazyme) was added, and the plate was incubated at 37 °C and 5% CO2 for 1 h. Absorbance was measured at 450 nm on a microplate reader (TECAN).

For EdU proliferation assay, EOC cells were plated at a density of 6000 cells per well in 96-well plates 1 day before treatment. Protocols were performed according to the of Cell-Light EdU Apollo567 *In Vitro* Kit (RiboBio).

### Chemosensitivity assay

Lentivirus infected and siRNA transfected EOC cells were plated at a density of 6000–8000 cells per well in 96-well plates. A series of cisplatin (Sigma-Aldrich) concentrations (0, 5, 10, 15, and 20 μM) were added. After 48 h of treating, CCK8 was used to detect surviving cells. IC50 was graphically calculated by Graphpad 8.0.

### Immunofluorescence (IF)assay

γH2AX foci presents the degree of DNA double strand breaks, which could be used to evaluate cell DNA damage [17]. EOC cells were seeded in confocal dishes and treated with cisplatin (5 μM) for 6 h. Cells were fixed in 4% paraformaldehyde and treated with 0.5% of Triton X-100 for 20 min and blocked in 1% Bovine Serum Albumin. Then cells were incubated with the first antibody in 4 °C for overnightand then incubated with the secondary antibody at room temperature for 1 h. The antibodies used for the IF assay were listed in Supplementary Table 3. Finally, cell nucleus was stained with DAPI (Merck). Images were captured by using Zeiss microscope.

### Cell cycle assay and apoptosis assay

Cells were plated in 6-well plates, and respectively treated with cisplatin (5 μM) and phosphate buffer saline (PBS) for 48 h. For the cell cycle analysis, 1 × 10^6^ cells and the cultural supernatant were collected, centrifuged, and washed. Cells were fixed with 75% cold ethanol for 24 h at − 20 °C. Next, the fixed cells were stained in 500 μl propidium oxide staining solution at room temperature for 15 min in the dark. For the apoptosis assay, 2× 10^4^ cells and the cultural supernatant were collected. Then, 5 μl of FITC Annexin V and 5 μl of propidium iodide (BD Biopharmingen) were added to the collected cells and suspending in 300 μl binding buffer for 15 min in the dark. Flow cytometry was performed to analyze cell cycle and cell apoptosis.

### Immunohistochemistry (IHC) assay

Firstly, surgical samples were pretreated in 10% formaldehyde for fixed. Staining was performed by the protocol as we previously reported [16]. The antibodies used for the IHC assay were listed in Supplementary Table 3.

### RNA binding protein immunoprecipitation (RIP)-qPCR

The RIP assay was performed using a MagnaRIP RNA-Binding Protein Immunoprecipitation Kit (Millipore). Briefly, the cell lysates were incubated with beads coated with 5 μg of antibodies with rotation at 4 °C overnight. Then, the RNA-protein-magnetic beads complexes were washed and eluted with proteinase K digestion buffer. Immunoprecipitated RNA was finally extracted by phenol-chloroform RNA extraction methods. Finally, enriched RNA was determined by qPCR and normalized to the input. The antibodies used for the RIP assay were listed in Supplementary Table 3.

### Chromatin immunoprecipitation (ChIP)-qPCR

The ChIP assay was performed using a Chromatin Immunoprecipitation Kit (Millipore) according to the manufacturer’s instructions. EOC cells were cross-linked with 1% formaldehyde; quenched with glycine at room temperature. Then, cells were collected, washed, and resuspended in lysis buffer. Then cross-linked DNAs were fragmented with 6% energy, 30s for 6 cycles. The sonicated chromatin solution was incubated with beads coated with 5 μg of antibodies with rotation at 4 °C for overnight. Immunoprecipitated DNA was purified and analyzed by qPCR and agarose gel electrophoresis assays. The specific primers were listed in Supplementary Table 2, and the antibodies used for ChIP assay were listed in Supplementary Table 3.

### Luciferase reporter assay

The luciferase reporters respectively containing the wild-type (WT) and mutated-type (Mut) sequences of JAK2 3′UTR (Chr12:5126686–5,127,015) were synthesized by Genechem (Shanghai, China). Luciferase reporters, respectively containing the WT and Mut sequences of ALKBH5 promotor (Chr17:18181828–18,181,968) were synthesized by Tsingke (Nanjing, China). Cells were seeded in a 24-well plate, and respectively transfected with the WT/Mut reporters. Luciferase assay was performed with Luciferase Kit (Promega) under the manufacturer’s instructions. The luciferase activity was measured by BERTHOL chemiluminescence measuring instrument (Centro XS LB 960). The sequences of the plasmids in the luciferase reporter assay were shown in Supplementary Table 5.

### Animal studies

The animal studies were performed in accordance with the institutional ethics guidelines for animal experiments approved by the animal management committee of Nanjing Medical University. About 5× 10^6^ cells were injected subcutaneously into the axilla of the female athymic BALB/C nude mice (4 week-old, 18–20 g). When the average tumor size reached approximately 100mm^3^ (after 1 week), mice were then randomized into two groups and treated with cisplatin (5 mg/kg) or normal saline (NS) weekly. Tumor width (W) and length (L) was measured every week, and the volume (V) of the tumor was calculated as the formula V = (W^2^ × L)/2. Every group was treated by 6 cycles of cisplatin/NS treatment until mice were euthanized, and tumors were removed for further study.

### Dot blot assay

mRNA was isolated from total RNA under the protocols of Kit (Promega). The dot blot assay was performed according to the bio-protocol database (https://en.bio-protocol.org/e2095). Briefly, 150/300 ng isolated mRNA was then spotted onto a Hybond-N+ membrane and cross-linked by a UV cross-linker. Methylene blue was used to interact with mRNA, and as the loading control, images were acquired. After washing, the membrane was washed and incubated first in blocking buffer and then with an anti-m6A antibody (1:250) at 4 °C fot overnight. Then, the membrane was rewashed and incubated with an anti-rabbit antibody (1:10000). Eventually, the membrane was exposed to Hyperfilm ECL (Bio-Rad), and images were acquired. The antibodies used for dot blot assay were listed in Supplementary Table 3.

### Actinomycin-D (act-D) assay

Cells were plated in 6-well plates and treated with actinomycin D (5 μg/mL, Med Chem Express) respectively for 0, 2, 4, and 6 h. Total RNA was then extracted and quantified by qPCR. The gene expression at the indicated time was calculated and normalized by GAPDH. The degradation rate of mRNA was estimated by the linear regression analysis.

### m6A-modified RNA immunoprecipitation sequencing (MeRIP-seq) and MeRIP-qPCR

Intact mRNA was first isolated from total RNA samples using mRNA Isolation Kit according to the manufacturer’s protocol (Promega), and the amount of purified mRNA was greater than 5 μg. The Magna MeRIP™ m6A Kit (Millipore) was then used for MeRIP according to the manufacturer’s instructions. Briefly, the isolated mRNA was chemically fragmented into 200-nucleotide-long fragments by incubation at 94 °C for 5 min, and the size of the fragmented mRNA was confirmed by Agilent 2100 Bioanalyzer (Agilent, CA, USA). Then, m6A-methylated mRNAs were immunoprecipitated with the m6A-antibody. The major procedures including immunoprecipitation, washing, and elution. Then eluted RNA and MeRIPed RNA were analyzed by deep sequencing on an Illumina Novaseq™ 6000 platform at the LC-BIO Bio-techltd (Hangzhou, China) following the vendor’s recommended protocol. The immunoprecipitated samples were also analyzed by MeRIP-qPCR. The specific primers are provided in Supplementary Table 2.

### Statistical analysis

All data and error bars are presented as the mean ± SDs from at least three independent experiments. The two-tailed Student’s t-test were performed to evaluate differences between two independent groups. The Graphpad 8.0 software was used to analyze the data. The indicated *p*-values (*p < 0.05, ** *p* < 0.01, *** *p* < 0.001 and **** *p* < 0.0001) were considered statistically significant.

## Results

### ALKBH5 promotes cell resistance to cisplatin in vivo and in vitro

**Fig. 1:**
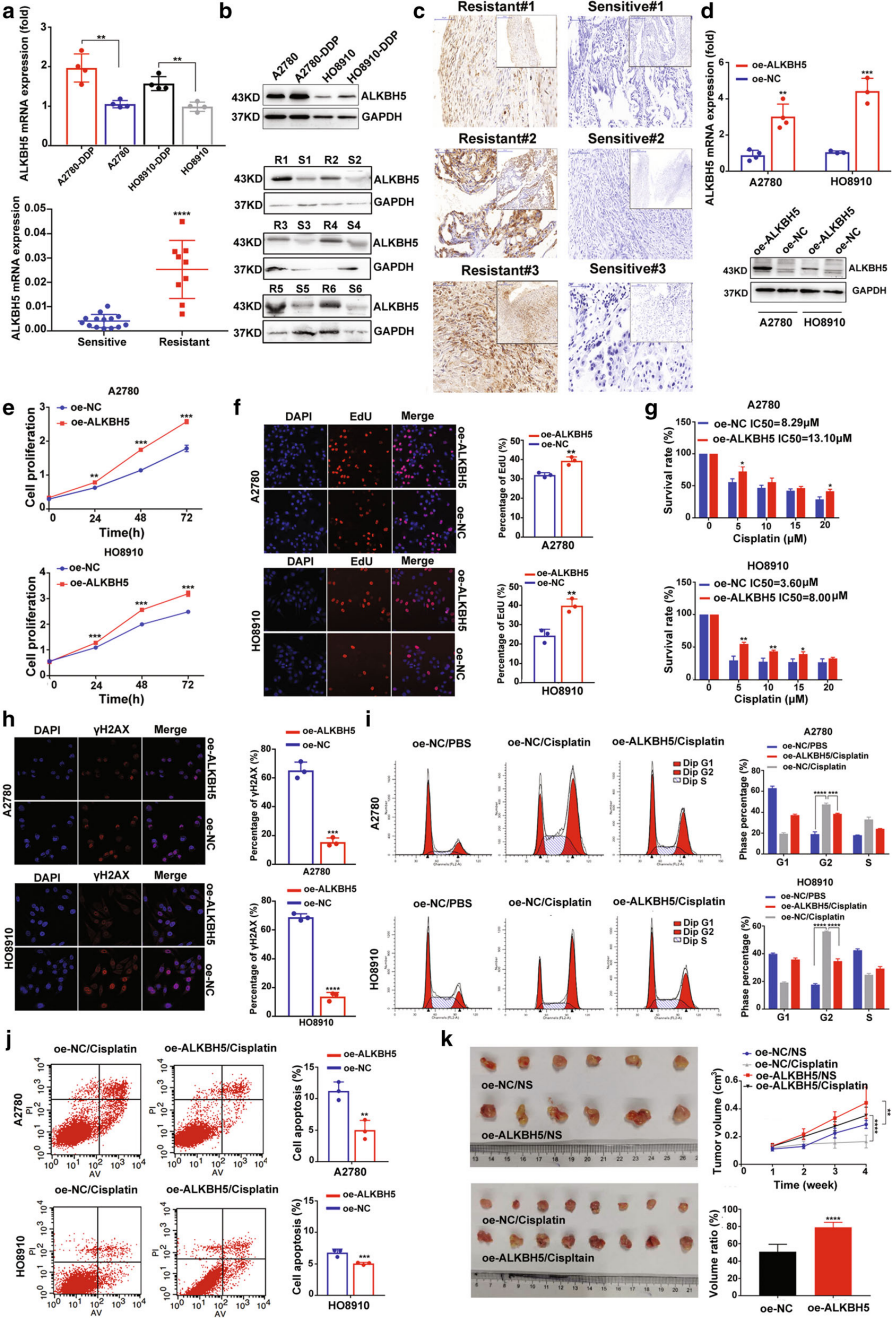
ALKBH5 overexpression promotes EOC cell proliferation and resistance to cisplatin in vitro and in vivo. (**a**) ALKBH5 mRNA expression is up-regulated in cisplatin-resistant EOC cells (upper); ALKBH5 mRNA expression is up-regulated in platinum-resistan EOC samples (down). (**b**) Results of the western-blot assays shows ALKBH5 was up-regulated both in cisplatin-resistant EOC cells (upper) and platinum-resistant EOC samples (down). (**c**) Expression of ALKBH5 protein in platinum-resistant (left) and platinum-sensitive (right) EOC tissues by IHC assay. (**d**) The transfection efficiency of oe-ALKBH5 lentivirus in A2780 and HO8910. (**e** and **f**) CCK8 and EdU proliferation assays confirm that ALKBH5 overexpression promotes cell proliferation in EOC cells. (**g**) The chemosensitivity assay shows that the IC50 of cisplatin is higher in EOC cells with ALKBH5 overexpression. (**h**) The IF assay shows that γH2AX expression is reduced in EOC cells with ALKBH5 overexpression after cisplatin treatment (5 μM, 6 h). (**i**) The cell cycle assays show that ALKBH5 overexpression can attenuate blocking in the G2/M phase induced by cisplatin (5 μM, 48 h). (**j**) The apoptosis assay shows that ALKBH5 overexpression can decrease the percentage of cell apoptosis induced by cisplatin (5 μM, 48 h). (**k**) The animal study shows that ALKBH5 overexpression promotes tumor growth and chemoresistance to cisplatin in vivo

By analysing the expression of m6A-related methyltransferases and demethyltransferase, ALKBH5 expression was found up-regulated both in cisplatin-resistant EOC cells (Fig. 1A and B, Supplementary Figs. 1A). Meanwhile, ALKBH5 was also found up-regulated in platinum-resistant EOC samples (Figs. 1A-C). The descriptive analysis of ALKBH5 mRNA expression and patients’ clinical characteristics was showed in Supplementary Table 6. CCK8 and EdU assays showed that ALKBH5 overexpression significantly promoted cell proliferation (Fig. 1D-F), while the opposite effect was observed after knocking down ALKBH5 both in cisplatin-sensitive (Supplementary Fig. 1B-D) and cisplatin-resistant cells (Supplementary Fig. 1E-G). The chemosensitivity assay showed an increased IC50 in EOC cells with ALKBH5 overexpression (Fig. 1G). In contrast, ALKBH5 knockdown weakened the chemoresistance in cisplatin-resistant EOC cells (Supplementary Fig. 1H). Moreover, ALKBH5 overexpression could relieve the DNA damage induced by cisplatin, whereas ALKBH5 knockdown aggravated cisplatin-induced DNA damage (Fig. 1H and Supplementary Fig. 1I). Furthermore, cell cycle analysis showed that ALKBH5 overexpression significantly relieved the blocking effect in the G2/M phase induced by cisplatin (Fig. 1I). Apoptosis analysis also showed that ALKBH5 overexpression reduced cisplatin-induced cell apoptosis (Fig. 1J). Consistently, animal studies showed that ALKBH5 overexpression promoted EOC tumor growth and resistance to cisplatin in vivo (Fig. 1K).

### ALKBH5-HOXA10 loop maintains ALKBH5 and HOXA10 overexpression in EOC

**Fig. 2:**
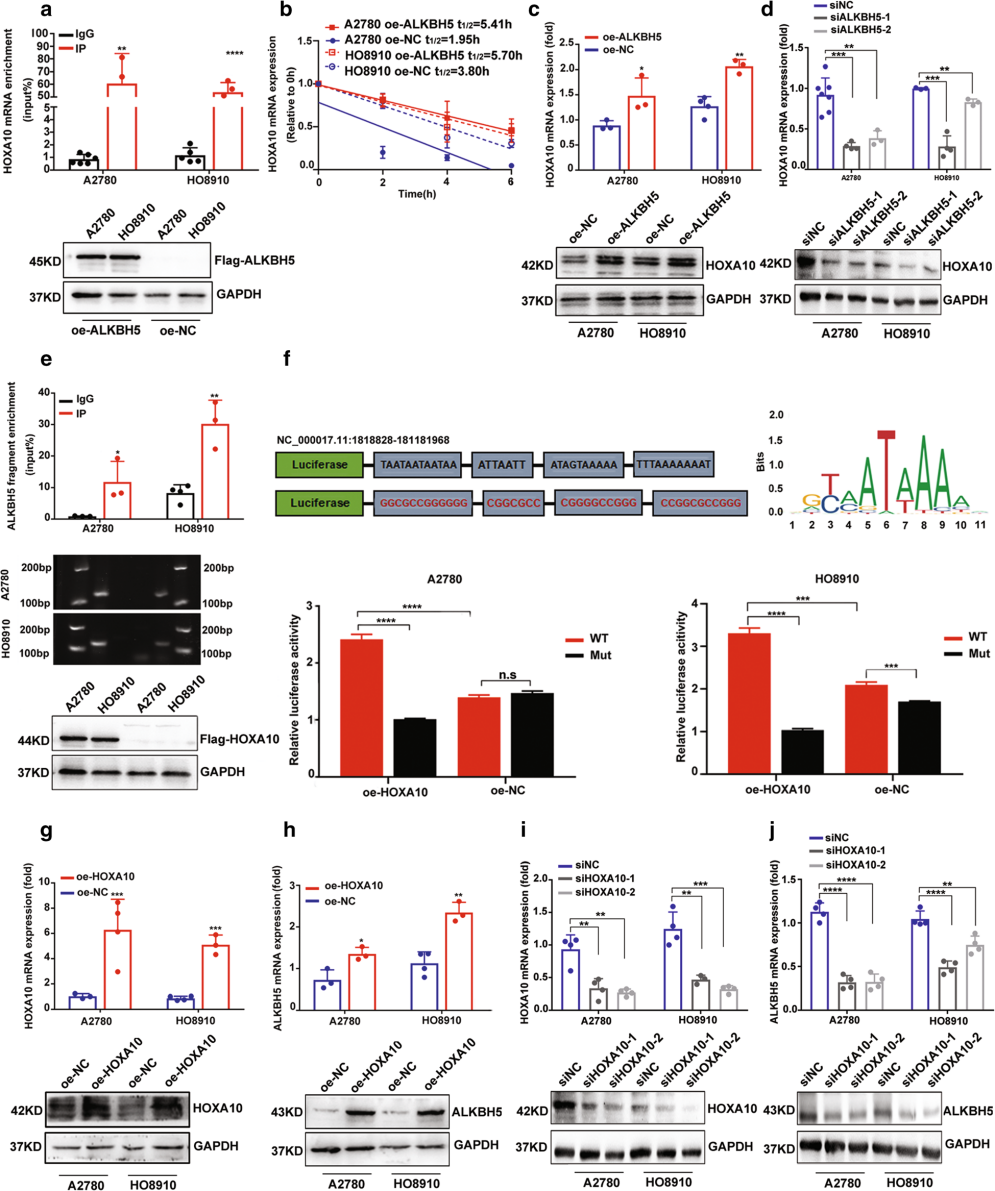
ALKBH5-HOXA10 loop maintains ALKBH5 and HOXA10 overexpression in EOC. (**a**) The results of the RIP-qPCR assay show that HOXA10 mRNA is enriched in Flag-specific antibody in EOC cells on transfection with oe-ALKBH5 using Flag-tag. (**b**) The Act-D assay shows that ALKBH5 overexpression can maintain HOXA10 mRNA stability in EOC cells. (**c** and **d**) ALKBH5 regulates HOXA10 expression in EOC. (**e**) The ChIP-qPCR assay suggests that the ALKBH5 promoter fragment is enriched in HOXA10 (top), and the AGE assay validates the sonicated products and immunoprecipitated DNA of the ChIP assay (middle). EOC cells are transfected with HOXA10-Flag-tag (bottom). (**f**) Based on the TF binding motif of HOXA10 predicted by the JASPAR database (top), the luciferase reporter assay is conducted, and the results show that HOXA10 could interact with the region containing TAAA of ALKBH5 promoter (bottom). (**g**) The transfection efficiency of oe-HOXA10 lentivirus in A2780 and HO8910. (**h**) ALKBH5 is up-regulated in EOC cells with HOXA10 overexpression. (**i**) The transfection efficiency of specific siRNAs targeting HOXA10. (**j**) ALKBH5 expression is down-regulated by knocking down HOXA10 in EOC cells

To explore the molecular mechanism of ALKBH5-mediated EOC resistance to cisplatin, RNA sequencing (RNA-seq) was performed. The results of functional annotations of RNA-seq via Gene Ontology (GO) analysis showed enhanced DNA repair in EOC cells with ALKBH5 upregulation (Supplementary Fig. 2, Additional file 1). Further, RNA-seq demonstrated that homeobox A10 (HOXA10) was highly upregulated in EOC cells with ALKBH5 overexpression (log2 fold change (FC)| = 2.52). Our previous studies have demonstrated that HOXA10 influences EOC cell proliferation, metastasis, invasion, and differentiation [18–20]. RIP-qPCR assay indicated that HOXA10 mRNA was significantly enriched in Flag-specific antibody in EOC cells transfected with oe-ALKBH5 using Flag-tag, suggesting that HOXA10 mRNA could bind to ALKBH5 (Fig. 2A). According to its transcript expression change and the potential regulatory mechanism, we speculated that ALKBH5 might mediate HOXA10 upregulation by maintaining the stability of HOXA10 mRNA. Results of the Act-D assay indicated that the half-life period of HOXA10 mRNA was significantly increased with ALKBH5 overexpression (Fig. 2B).

Analysis of the correlation between ALKBH5 and HOXA10 in 57 EOC tissues and 426 samples of The Cancer Genome Atlas (TCGA) database showed that mRNA expression of HOXA10 was positively correlated with ALKBH5 expression (Supplementary Fig. 3A). Consistently, HOXA10 expression was found to be regulated by ALKBH5 up-regulation or down-regulation in EOC cells (Fig. 2C and D). Considering the role of HOXA10 as a transcription factor (TF), we analyzed data from the JASPAR database and unexpectedly found probable binding motifs with HOXA10 in the ALKBH5 promotor region. The sonicated chromatin solution of EOC cells was immunoprecipitated with an anti-Flag antibody and IgG control antibody. Results of the ChIP-qPCR showed that the Flag-specific antibody was significantly enriched in DNA fragments contained in the ALKBH5 promoter region (Fig. 2E). By validating the potential transcription binding motifs of HOXA10 and ALKBH5 predicted by the JASPAR database, we constructed luciferase reporting gene plasmids encoding WT and Mut specific sequences of ALKBH5 promoter regions. The luciferase reporter assay results suggested that HOXA10 could be a TF to interact with the TAAA region of the ALKBH5 promoter (Fig. 2F). Consequently, ALKBH5 expression was confirmed to be up-regulated in EOC cell with HOXA10 overexpression, while down-regulated after knocking down HOXA10 expression (Fig. 2G-J). These data suggested that HOXA10 might play as an upstream TF of ALKBH5 and contribute to the upregulation of ALKBH5 in EOC. Our findings revealed that the ALKBH5-HOXA10 regulation loop steadily maintained the overexpression of both ALKBH5 and HOXA10 in EOC.

### HOXA10 overexpression promotes the proliferation and cisplatin-resistance of EOC in vivo and in vitro

**Fig. 3:**
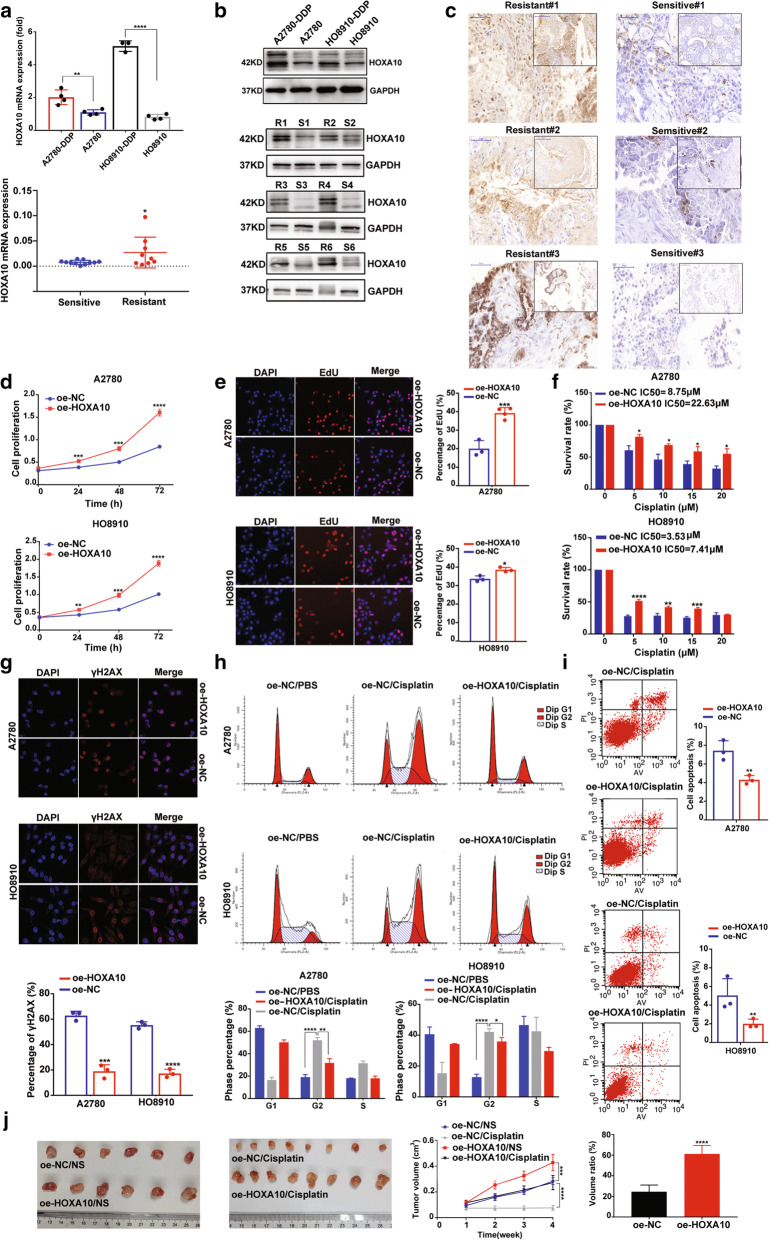
HOXA10 overexpression promotes EOC cell proliferation and cisplatin resistance in vitro and in vivo. (**a**) HOXA10 mRNA is upregulated in cisplatin-resistant EOC cells and platinum-resistant EOC samples. (**b**) Results of the western-blot assays shows HOXA10 was up-regulated both in cisplatin-resistant EOC cells (upper) and platinum-resistant EOC samples (down). (**c**) Expression of HOXA10 protein in platinum-resistant (left) and platinum-sensitive (right) EOC tissues by IHC assay. (**d** and **e**) CCK8 and EdU proliferation assays confirm that HOXA10 overexpression promotes cell proliferation in EOC cells. (**f**) The chemosensitivity assay demonstrates higher IC50 of cisplatin in EOC cells with oe-HOXA10 transfection. (**g**) The IF assay shows that γH2AX foci is decreased in EOC cells with HOXA10 overexpression after cisplatin treatment (5 μM, 6 h). (**h**) The cell cycle assays show that HOXA10 overexpression can alleviate the blocking in the G2/M phase induced by cisplatin (5 μM 48 h). (**i**) The apoptosis assays show that HOXA10 overexpression can lower the percentage of cell apoptosis induced by cisplatin (5 μM, 48 h). (**j**) The animal study shows that HOXA10 overexpression promotes cell proliferation and resistance to cisplatin in vivo

Given that ALKBH5 was confirmed as a factor that promotes EOC resistance to cisplatin and forms a positive regulation loop with HOXA10, we speculated that HOXA10 might also promote cisplatin resistance in EOC. The results showed that HOXA10 expression was upregulated in A2780-DDP and HO8910-DDP cells (Fig. 3A and B). Additionally, HOXA10 was confirmed to be overexpressed in platinum-resistant EOC samples (Fig. 3A-C). The descriptive analysis of HOXA10 mRNA expression and clinical characteristics of 57 EOC patients was showed in Supplementary Table 7. The results of proliferation asasays confirmed that HOXA10 overexpression significantly promoted cell proliferation (Fig. 3D and E), while an opposite effect was exerted after knocking down HOXA10 in both cisplatin-sensitive (Supplementary Fig. 3B and 3C) and cisplatin-resistent EOC cells (Supplementary Fig. 3D-3F). Further, HOXA10 overexpression promoted cisplatin resistance and attenuated the DNA damage in cisplatin-sensitive EOC cells (Fig. 3F and G). In contrast, HOXA10 knockdown weakened cisplatin resistance and aggravated DNA damage in cisplatin-resistant EOC cells (Supplementary Fig. 3G and 3H). In cell cycle analysis, HOXA10 overexpression significantly altered the inhibition effect of cisplatin in the G2/M phase (Fig. 3H). The apoptosis analysis results showed that HOXA10 overexpression inhibited EOC cell apoptosis induced by cisplatin (Fig. 3I). The xenograft model demonstrated that HOXA10 promoted tumor growth and resistance to cisplatin (Fig. 3J).

### ALKBH5 erases m6A modifications of JAK2 mRNA and maintains JAK2 mRNA expression by lowering YTHDF2-mediated mRNA degradation

**Fig. 4:**
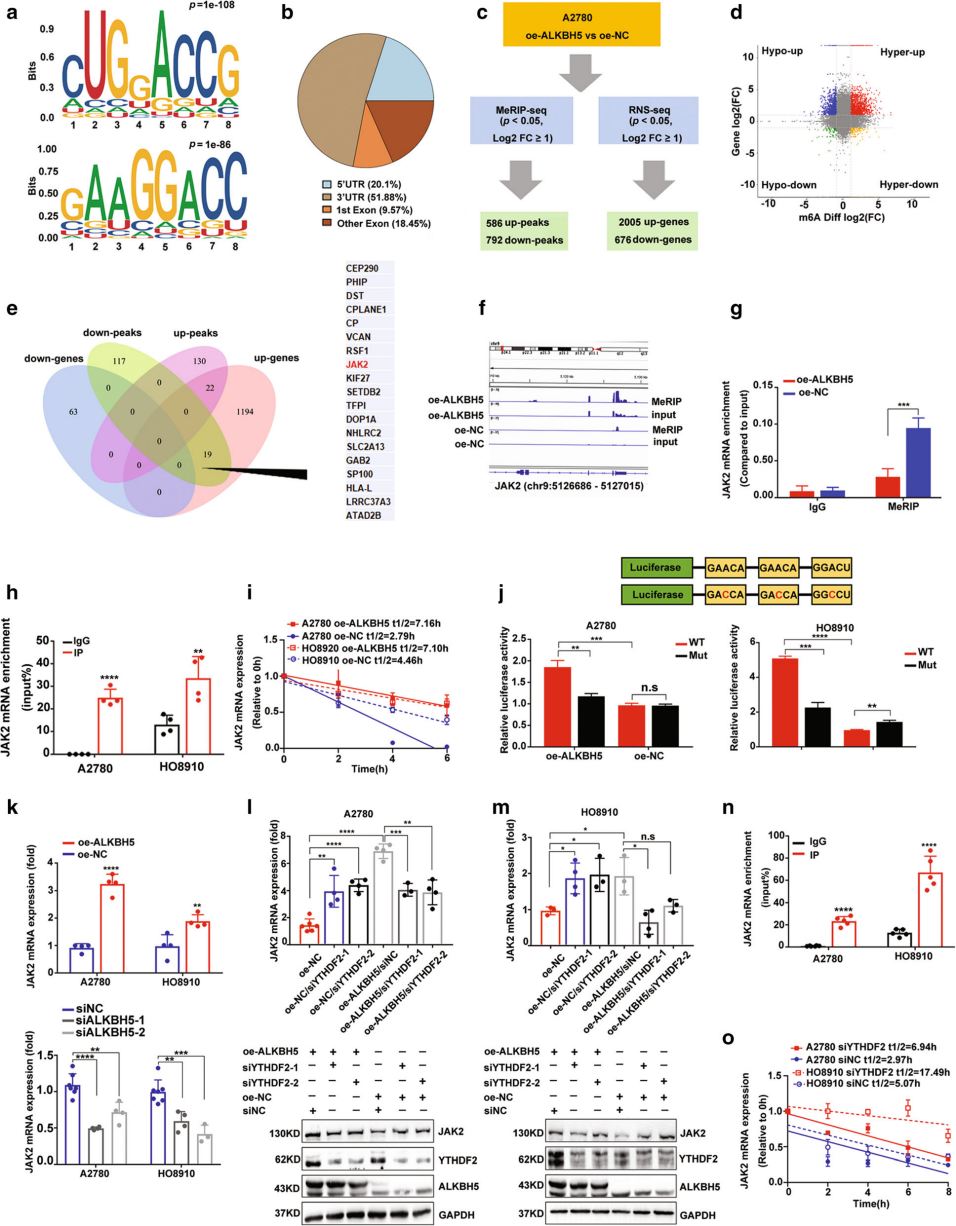
ALKBH5 erases m6A modifications of JAK2 mRNA and maintains JAK2 mRNA expression by lowering YTHDF2-mediated mRNA degradation. (**a**) The two differentially enriched m6A-modification motif in the immunopurified RNA in A2780. (**b**) Distribution of regulated m6A peaks in mRNA is detected by MeRIP-seq after ALKBH5 overexpression. (**c**) Schematic of downstream analysis for MeRIP-Seq and RNA-seq. (**d**) The quadrantal diagram graph displays the transcripts with different m6A peaks and regulated gene expression based on MeRIP-seq and RNA-seq. (**e**) The Venn diagram shows the genes detected by MeRIP-seq and RNA-seq; the 19 candidate target genes of ALKBH5 are shown on the right. (**f**) m6A abundances in JAK2 mRNA transcripts in cells with ALKBH5 overexpression (MeRIP and input) and in negative control (MeRIP and input). m6A regulation is calculated as the ratio of m6A abundances of MeRIP to input (log2FC = − 2, *p* = 0.03). (**g**) MeRIP-qPCR confirms that ALKBH5 down-regulates the m6A peak in 3’UTR of JAK2 mRNA. (**h**) RIP-qPCR confirmed JAK2 mRNA binding to ALKBH5. (**i**) The Act-D assay shows an increased lifespan of JAK2 mRNA after ALKBH5 overexpression. (**j**) Relative luciferase activity of the wild-type or mutant JAK2 3′UTR luciferase reporter in EOC cells with ALKBH5 overexpression and the negative control. (**k**) ALKBH5 positively regulates JAK2 mRNA expression in EOC cells. (**l** and **m**) YTHDF2 remarkably regulates JAK2 expression in A2780 and HO8910. (**n**) RIP-qPCR confirms YTHDF2 binding to JAK2 mRNA. (**o**) Increased lifespan of JAK2 mRNA after YTHDF2 silencing

Given that ALKBH5 was identified as one of the m6A erasers, we performed the m6A dot blot assay and found that ALKBH5 upregulation in EOC cells decreased the m6A modification level of mRNA (Supplementary Fig. 4A). We further analyzed the targeted m6A-modified genes of ALKBH5 in EOC using RNA-Seq and MeRIP-Seq analysis. The MeRIP-Seq results validated the differentially explored m6A modification motif in the m6A-immunopurified RNA in A2780 with ALKBH5 overexpression and the negative control (Fig. 4A). Further, 51.88% of the m6A abundance was differentially enriched in three prime untranslated regions (3’UTRs) of transcripts (Fig. 4B). MeRIP-seq revealed that ALKBH5 upregulation resulted in increased abundance of m6A peaks in 586 transcripts and decreased abundance in 792 transcripts (|log2 FC| ≥ 1, *p <* 0.05) (Fig. 4C and Additional file 2). RNA-seq also revealed that there were 2005 upregulated and 676 downregulated genes in A2780 with ALKBH5 upregulation (|log2 (FC)| ≥ 1, *p <* 0.05) (Fig. 4C and Additional file 3). The quadrantal diagram graph displayed transcripts with a different abundance of m6A peaks and regulated gene expression (Fig. 4D). ALKBH5 mediated m6A demethylation of mRNA, which could influence mRNA metabolism processes such as stabilization and degradation. Thus, we focused on genes with both changes in m6A modification and mRNA expression. There were 19 genes in cells with ALKBH5 upregulation that exhibited not only decreased abundance of m6A peaks (log2 FC ≤ − 2, *p* < 0.05) but also significantly discrepant expression regulation (log2 FC ≥ 2, *p* < 0.05) (Fig. 4E and Table 1). This suggested that these 19 genes might be contained in the target m6-modified genes regulated by ALKBH5. Among them, m6A abundance in the 3’UTR region of JAK2 mRNA (Chr12: 5126686–5,127,015) was notably significantly decreased after ALKBH5 upregulation in A2780 (log2 FC = − 2, *p* = 0.03) (Fig. 4F). The JAK2/STAT3 signaling pathway has been widely demonstrated to be involved in tumor growth and chemoresistance in various malignancies [21]. Thus, we speculated that ALKBH5 overexpression might promote EOC cell proliferation and resistance to cisplatin by targeting JAK2 and activating the JAK2/STAT3 signaling pathway in the m6A-dependent manner. The results of MeRIP-qPCR also confirmed that ALKBH5 overexpression decreased the m6A modification abundance of the 3’UTR region of JAK2 mRNA in A2780 (Fig. 4G). The results of RIP-qPCR assay showed that JAK2 mRNA expression was higher in the Flag-specific antibody than in the IgG antibody (Fig. 4H). We then performed the Act-D assay to investigate whether ALKBH5 expression affected the stability of JAK2 mRNA. As shown in Fig. 4I, JAK2 mRNA expression was highly stable in EOC cells with ALKBH5 overexpression. The distribution of m6A is usually embedded within the consensus sequence 5′-DRACH′ (D = G/A/U, *R =* A/G, H = not G). Based on the results of MeRIP-seq, we mutated three “DRACH” motifs of JAK2 3’UTR (Chr12: 5126686–5,127,015) to construct the WT/Mut luciferase reporters. Moreover, results of the luciferase reporter assay showed that ALKBH5 recognized and was bound to the m6A-motif in the 3’UTR region of JAK2 mRNA and promoted *JAK2* expression (Fig. 4J). Consistent with the above findings, JAK2 mRNA expression was up-regulated in cell with ALKBH5 overexpression, while down-regulated by knocking down ALKBH5 in EOC cells (Fig. 4K). Previous studies have identified YTHDFs, including YTHDF1/2/3, as a family of m6A readers that target thousands of mRNA transcripts by distinctly recognizing the m6A motif. In the cytosol, YTHDF1 enhances the translation of its targets by interacting with initiation factors and facilitating ribosome loading. YTHDF3 affects the translation of its target mRNAs along with YTHDF1. Meanwhile, YTHDF2 helps promote mRNA degradation of m6A-modified mRNA [22]. In the present study, we found a lower m6A abundance of JAK2 mRNA in EOC cell with ALKBH5 overexpression, whereas JAK2 mRNA expression was upregulated. Considering the above results, we speculated that YTHDF2 might mediate m6A-JAK2 degradation. We then used specific siRNAs to knock down YTHDF2 expression in EOC cells. The results showed that knocking down YTHDF2 up-regulated JAK2 expression compared to the negative control. Meanwhile, knocking down YTHDF2 expression could partly reduce JAK2 upregulation induced by overexpression of ALKBH5 (Fig. 4L and M). The RIP-qPCR assay showed that the YTHDF2-specific antibody caused a significantly higher increase in JAK2 mRNA enrichment compared to IgG (Fig. 4N). The Act-D assay confirmed that the level of JAK2 mRNA was more stable after YTHDF2 knockdown (Fig. 4O). Collectively, these findings indicated that ALKBH5-mediated JAK2 mRNA m6A demethylation inhibited YTHDF2-mediated mRNA degradation and maintained JAK2 mRNA expression.

**Table 1:** List of 19 genes selected by MeRIP-seq and RNA-seq

Gene name	RNA-seq		MeRIP-seq	
	diff. p	diff. log2fc	diff. p	diff. log2fc
NHLRC2	0.002	−2.83	0.001	2.49
SLC2A13	0.006	−2.16	0.001	2.50
TFPI	0.009	−2.09	0.000	2.72
SETDB2	0.009	−2.54	0.000	2.95
VCAN	0.012	−2.23	0.000	3.50
CPLANE1	0.018	−2.18	0.000	4.00
CP	0.019	−2.86	0.000	3.88
KIF27	0.019	−2.95	0.000	3.37
CEP290	0.024	−2.15	0.000	4.42
GAB2	0.025	−3.17	0.002	2.40
DST	0.025	−2.93	0.000	4.01
HLA-L	0.027	−2.92	0.005	2.23
PHIP	0.028	−2.20	0.000	4.38
SP100	0.030	−2.03	0.002	2.34
JAK2	0.035	−2	0.000	3.35
DOP1A	0.036	−2.12	0.000	2.74
ATAD2B	0.037	−2.76	0.008	2.04
HLA-L	0.045	−2.63	0.005	2.23
VCAN	0.047	−2.65	0.000	3.50
LRRC37A3	0.047	−2.87	0.007	2.17
RSF1	0.048	−2.14	0.000	3.43

In total 19 genes were found exhibited obvious decreased abundance of m6A peaks (log2 (FC) ≤ − 2, *p *< 0.05), and displayed significantly discrepant expression regulation (log2 (FC) ≥ 2, *p*<0.05)

### ALKBH5-HOXA10 loop promotes resistance to cisplatin by activating the JAK2/STAT3 signaling pathway in EOC

**Fig. 5:**
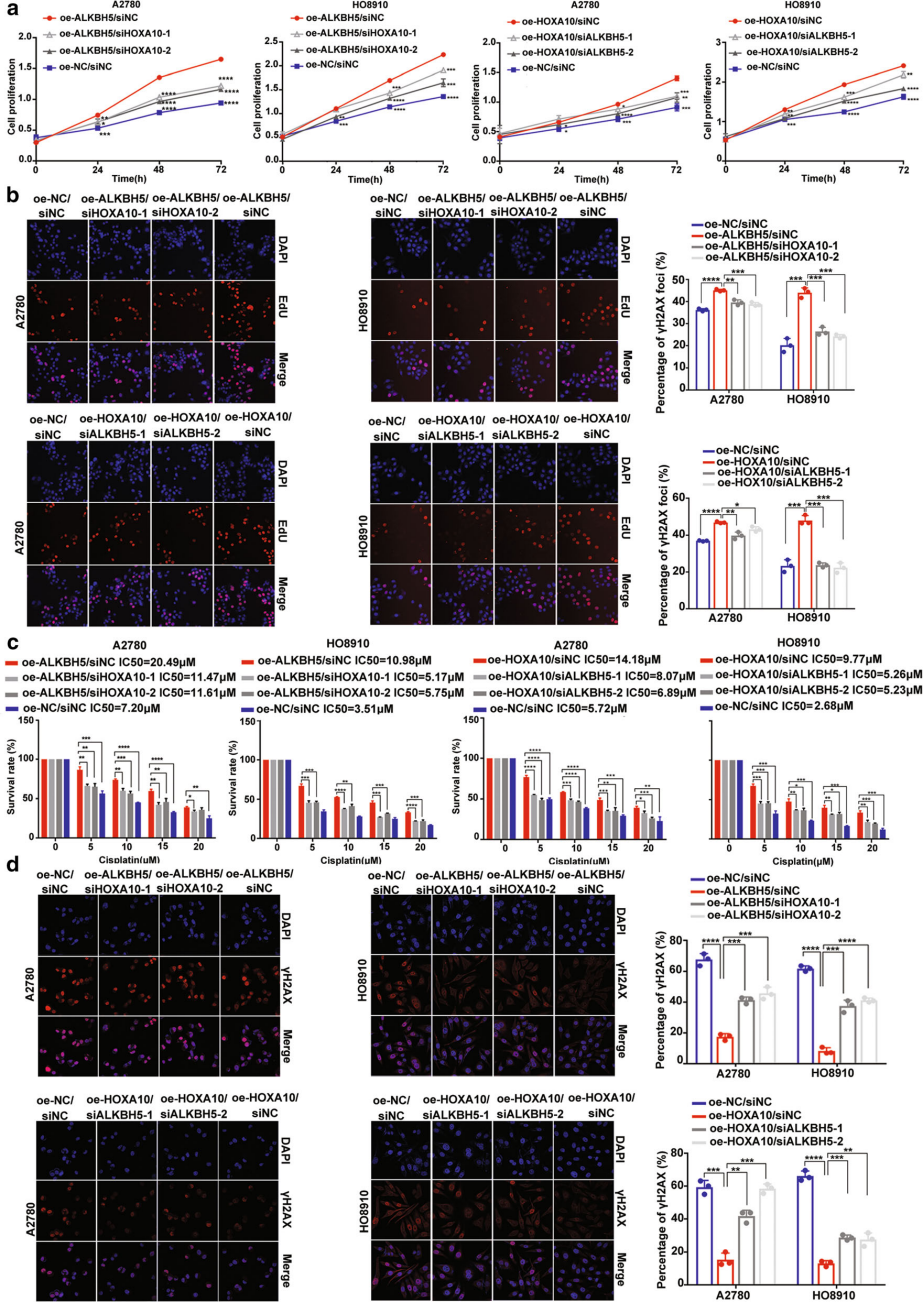
ALKBH5-HOXA10 loop steadily promotes cell proliferation and resistance to cisplatin. (**a** and **b**) CCK8 and EdU proliferation assays show that using specific siRNAs to respectively knock down HOXA10 or ALKBH5 expression in EOC cells with ALKBH5 or HOXA10 expression could significantly suppress cancer cell proliferation. (**c** and **d**) HOXA10 or ALKBH5 knockdown rescues resistance to cisplatin and DDR in EOC cells with ALKBH5- HOXA10 loop overexpression

**Fig. 6:**
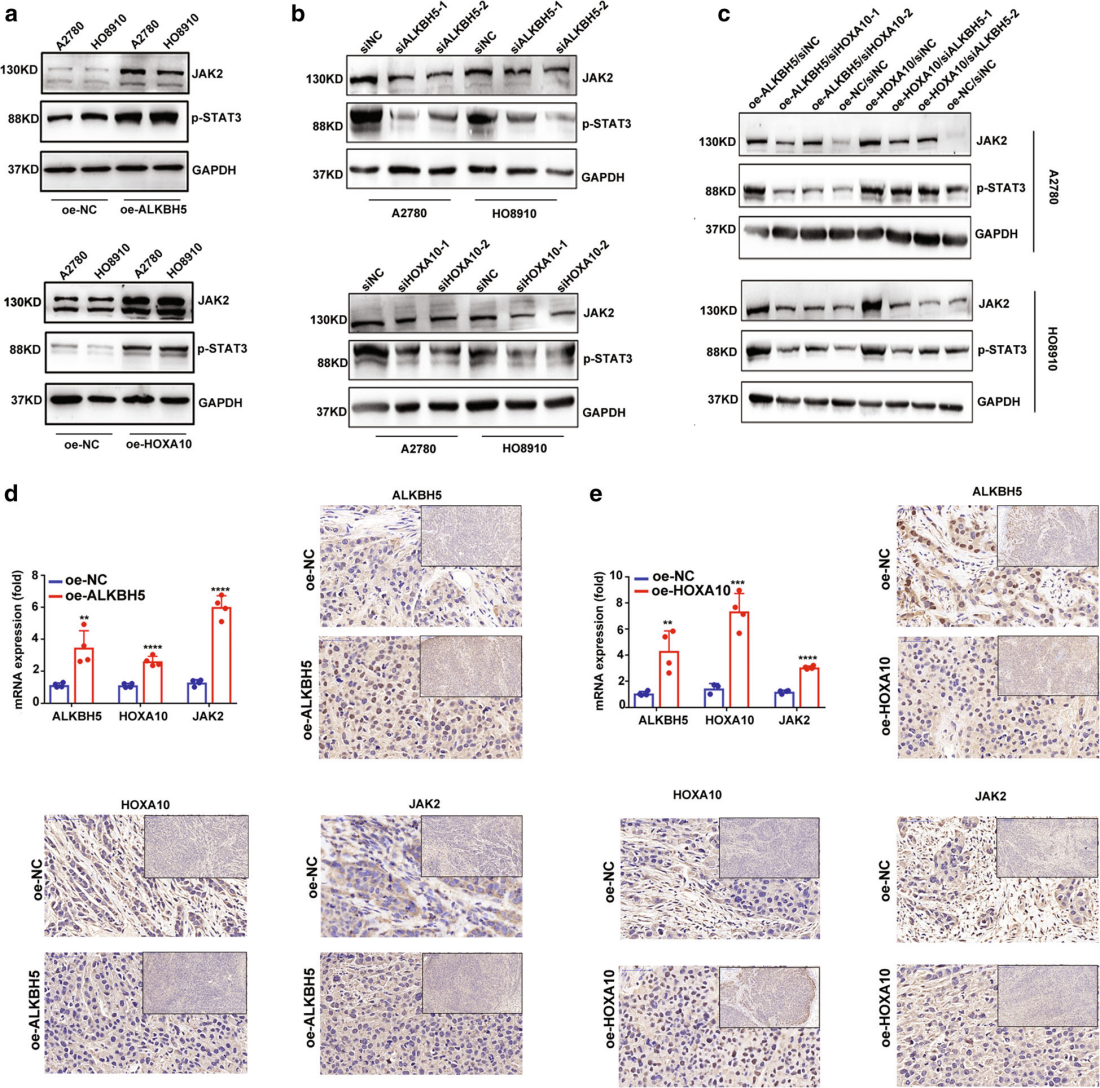
ALKBH5-HOXA10 loop overexpression activates the JAK2/STAT3 signaling pathway. (**a**) Upregulation of the ALKBH5-HOXA10 loop promotes JAK2 expression and the phosphorylation level of STAT3. (**b**) Downregulation of the ALKBH5-HOXA10 loop inhibits JAK2 expression and the phosphorylation level of STAT3. (**c**) Activation of the JAK2/STAT3 pathway can be rescued by knocking down ALKBH5 or HOXA10 respectively in cells with HOXA10 and ALKBH5 overexpression. (**d** and **e**) The IHC assays show ALKBH/HOXA10/JAK2 regulation in the xenograft model

We then investigated whether HOXA10 or ALKBH5 knockdown could rescue cisplatin resistance in EOC cell with ALKBH5 and HOXA10 overexpression. The results showed that knocking down HOXA10 in cells with ALKBH5 overexpression could partly inhibit cell proliferation, chemoresistance, and DNA damage response (DDR) (Fig. 5A-D). Similarly, ALKBH5 knockdown in cells with HOXA10 overexpression also partly relieved EOC cell proliferation, chemoresistance, and DDR (Fig. 5A-D). The results of correlation analyses in 57 EOC samples and TCGA database also showed that JAK2 mRNA expression was positively correlated with ALKBH5 or HOXA10 expression (Supplementary Fig. 4B and C). However, the correlation analysis results of HOXA10 and JAK2 expression based on the TCGA database showed no statistical significance. Further investigation confirmed that upregulation of the ALKBH5-HOXA10 loop could promote JAK2 expression and the phosphorylation level of STAT3, which represents activation of the JAK2/STAT3 signaling pathway (Fig. 6A). In contrast, knocking down ALKBH5 or HOXA10 decreased JAK2 expression and the phosphorylation level of STAT3, which indicates inhibition of the JAK2/STAT3 signaling pathway (Fig. 6B). Moreover, the activated JAK2/STAT3 pathway could be rescued by siHOXA10 and siALKBH5 in EOC cells with HOXA10 and ALKBH5 overexpression, respectively (Fig. 6C). Immunohistochemistry (IHC) assay of the xenograft tissues showed the same results (Fig. 6D and E). Collectively, these results support that JAK2/STAT3 signaling pathway might be involved in the regulation mechanism of the ALKBH5-HOXA10 loop in EOC. Then, we used two specific siRNAs to knock down JAK2 expression and found that JAK2 knockdown significantly rescued cell proliferation, cisplatin resistance, and DDR in cells with ALKBH5 or HOXA10 expression (Supplementary Fig. 5 and Supplementary Fig. 6). Furthermore, inhibition of JAK2/STAT3 signaling pathway using WP1066 effectively suppressed cancer cell proliferation, resistance to cisplatin, and DDR (Supplementary Fig. 7). Overall, these findings indicate that consistent overexpression of the ALKBH5-HOXA10 loop in EOC could promote tumor growth and chemoresistance by mediating the JAK2/STAT3 signaling pathway.

## Discussion

**Fig. 7:**
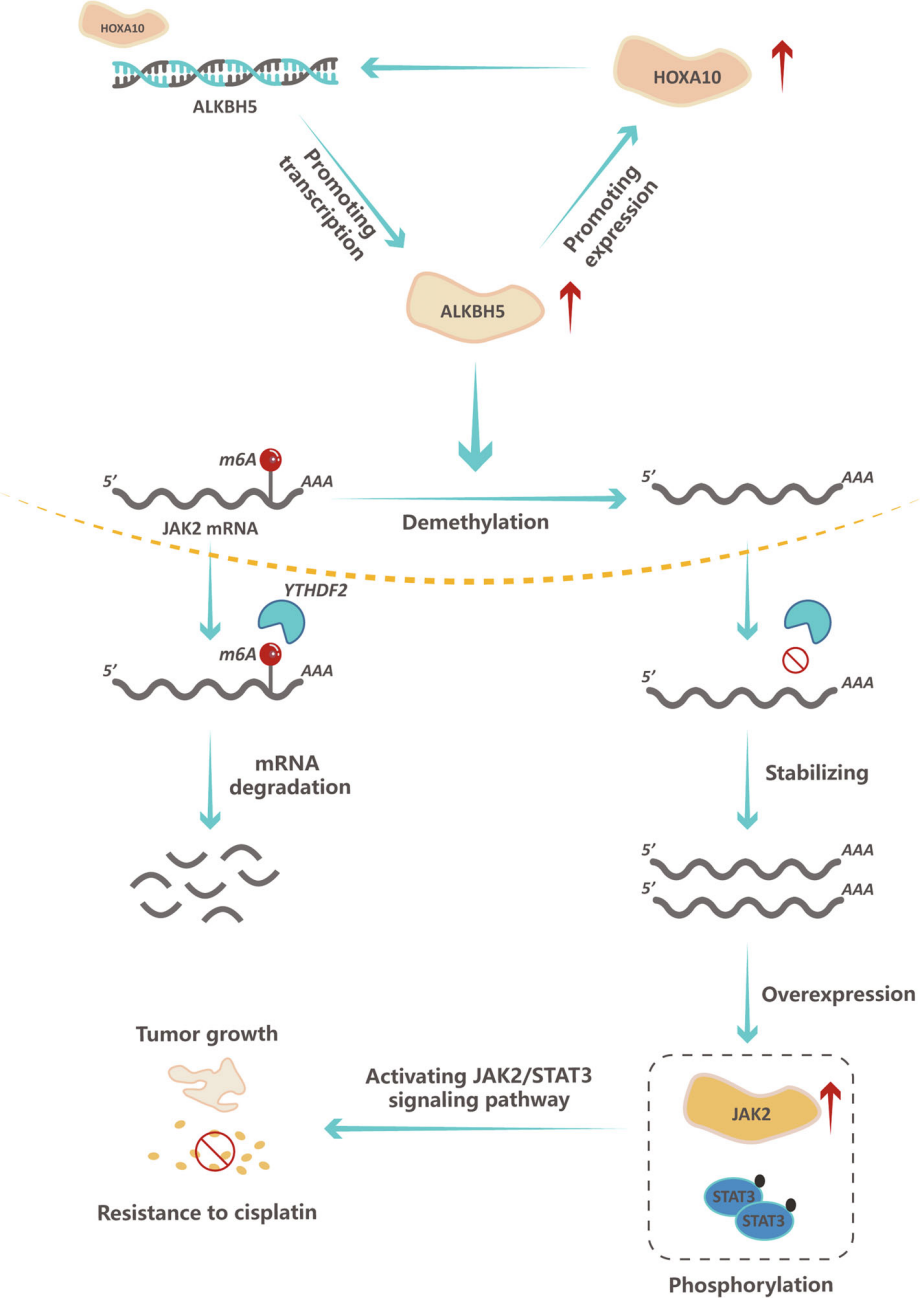
Graphic abstract. Overview of the present study

The role of m6A modification in cancer treatment resistance, particularly cisplatin chemotherapy in EOC, remains unclear. The present study found that ALKBH5 is upregulated in cisplatin-resistant EOC, promoting cell proliferation and chemoresistance in vivo and in vitro*.* Based on the results of RNA-seq, we identified the ALKBH5-HOXA10 positive regulation loop and then confirmed that HOXA10 could also promote cell proliferation and chemoresistance in vivo and in vitro. The MeRIP-seq results support that ALKBH5 reduces m6A modification in JAK2 mRNA and maintains JAK2 mRNA expression by reducing YTHDF2-mediated mRNA degradation. Furthermore, our findings revealed that ALKBH5-HOXA10 loop overexpression activates the JAK2/STAT3 signaling pathway, which, in turn, promotes cisplatin resistance in EOC (Fig. 7).

As one of the early adaptive mechanisms by which cells respond to environmental stress, m6A modification appears to be a promising target for cancer treatment. Previous studies have demonstrated the important roles of m6A-modulators in EOC, including MATTL3, ALKBH5, FTO, YTHDF1, and IGF2BP1 [13, 23–26]. These studies suggest that m6A modification significantly contributes to EOC initiation and progression. However, research on the role of m6A modification in EOC chemoresistance is lacking.

In recent years, ALKBH5 has been increasingly studied in various malignancies and has been found to play dual roles. Previous studies demonstrated that ALKBH5 acted as an oncogene in colon cancer, endometrial cancer, and renal cell carcinoma [27–29]. Further, ALKBH5 was found to suppress progression in pancreatic cancer and hepatocellular carcinoma [30, 31]. Moreover, ALKBH5 was also suggested to increase glioma stem cell radioresistance by regulating homologous recombination [32]. Meanwhile, Li et al. identified ALKBH5 to enhance treatment response to anti-PD-1 therapy in melanoma, colorectal, and potentially other cancers [33]. These studies indicated that ALKBH5 might be a potential target to overcome treatment resistance in cancer. The previous study confirmed that ALKBH5 was an oncogene in EOC [13]. Similar to our previous study, we have demonstrated that ALKBH5 promotes cell proliferation in both cisplatin-sensitive and cisplatin-resistant EOC cells. Importantly, we revealed that ALKBH5 was upregulated in cisplatin-resistant EOC cells and promoted cell resistance to cisplatin. Functional annotations based on the RNA-seq results suggested that ALKBH5 upregulation was associated with DNA repair processes in cancer cells. Subsequently, we found that γH2AX expression was significantly decreased in EOC cells with ALKBH5 overexpression. This suggested that ALKBH5 could enhance DNA repairs of DNA double-strand break induced by cisplatin. To our best knowledge, this study is the first to report ALKBH5 upregulation in cisplatin-resistant EOC and the consequent promotion of cell resistance from such upregulation.

HOXA10 is a member of the homeobox gene family that acts as a TF in embryonic development. Aberrant regulation of the homeobox family has been identified in various cancers [34–36]. Our previous studies have determined the critical role of HOXA10 in ovarian cancer [18, 37, 38], and we have been exploring the role of HOXA10 in female malignancies for a long time. However, studies on the role of HOXA10 in cisplatin-resistant EOC are lacking. The results of RNA-seq highlighted the importance of HOXA10, and further analysis revealed its crucial role in facilitating chemoresistance in EOC. Moreover, we identified ALKBH5-HOXA10 as a positive feedback loop that could steadily maintain each other’s upregulation. HOXA10 binding on the “TAAA” region of ALKBH5 promotor enhanced ALKBH5 transcription. Meanwhile, ALKBH5 could interact with HOXA10 mRNA and maintain its stabilization.

JAK2 functions as a prototypical kinase that phosphorylates STAT3, which activates the JAK2/STAT3 signaling pathway and promotes tumorigenesis and progression in several cancer types [39]. Activation of the JAK2/STAT3 signaling pathway was also proven to contribute to chemotherapy resistance in several malignancies, including EOC [40–42]. The m6A-dependent regulation of SOCS3 and JAK2 mediated by YTHDF1/YTHDF2 has been demonstrated in pluripotent stem cells [43]. However, to our best knowledge, no study has reported ALKBH5-mediated m6A demethylation of JAK2 to date. Based on the MeRIP-seq results, we found that ALKBH5 erased the m6A-modification of JAK2 mRNA 3’UTR. Thus, we further investigated the probable reader that mediated m6A-JAK2 mRNA metabolism and found that YTHDF2 could directly bind to JAK2 mRNA and interact with its m6A motif, which prevents the degradation of demethylated JAK2 mRNA. YTHDF2 is a well-established m6A reader that can weaken mRNA stability by interacting with m6A-containing mRNAs [44]. These findings suggested that ALKBH5 mediates JAK2 m6A demethylation in EOC cells, which maintains JAK2 mRNA expression by lowering YTHDF2-mediated mRNA degradation. These findings revealed the underlying mechanism of ALKBH5-mediated JAK2 regulation EOC. By further performing rescue assays, we subsequently confirmed the gene interaction of the ALKBH5-HOXA10 loop in cell proliferation and cisplatin resistance. Additionally, the critical role of ALKBH5-HOXA10 loop in mediating JAK2 upregulation was further demonstrated in cell proliferation and cisplatin resistance by down-regulating JAK2 expression or inhibiting JAK2/STAT3 signaling pathway.

This study has some limitations. As a well-known m6A demethyltransferase, ALKBH5 usually regulates the target gene expression in the m6A-dependent manner. Our study demonstrated that ALKBH5 could bind to HOXA10 and maintain mRNA expression. However, the MeRIP-seq results showed no differentially enriched m6A “peak” in HOXA10 mRNA after ALKBH5 overexpression. These findings suggest that ALKBH5 does not influence HOXA10 in a direct m6A-dependent manner. Thus, the exact molecular mechanism needs further investigations.

In summary, our findings revealed that m6A modification represents a novel mechanism of cisplatin resistance in EOC. ALKBH5 was upregulated in cisplatin-resistant EOC, and ALKBH5 overexpression promoted EOC cell proliferation and resistance to cisplatin in vivo and in vitro. HOXA10 was identified as a TF enhancing ALKBH5 transcription, and it could also be regulated by ALKBH5. Further, HOXA10 was found to play a role in EOC resistance to cisplatin. Further analysis of the m6A modification mechanism regulated by ALKBH5 showed that JAK2 is the m6-modified gene targeted by ALKBH5. ALKBH5 overexpression maintained JAK2 mRNA stability in a YTHDF2-mediated manner. Consistent upregulation of the ALKBH5-HOXA10 loop promoted EOC tumor growth and resistance to cisplatin by activating the JAK2/STAT3 signaling pathway in the m6A-dependent manner. Collectively, our results suggest that inhibition of the expression of the ALKBH5-HOXA10 loop represents a potential strategy to overcome cisplatin resistance in EOC.

## Supplementary Information

**Additional file 1.** Gene Ontology (GO) analysis of differentially expressed genes of RNA-seq.

**Additional file 2 **The differentially enriched m6A-peaks of MeRIP-seq (|log2 fold change (FC)| ≥1, *p*<0.05).

**Additional file 3 **The differentially expressed genes of mRNA-seq (|log2 (FC)| ≥ 1, *p* <0.05).

**Additional file 4.**

**Additional file 5.**

**Additional file 6.**

**Additional file 7.**

**Additional file 8.**

**Additional file 9.**

**Additional file 10.**

**Additional file 11 Supplementary Fig. 1** ALKBH5 downregulation inhibits cell resistance to cisplatin. (A) The mRNA expression of METTL3, METTL14, WTAP and FTO is validiated in cisplatin-sensitive and cisplatin-resistant EOC cells. (B) The transfection effieciency of specific siRNAs targeting ALKBH5 in cisplatin-sensitive EOC cells. (C and D) CCK8 and EdU proliferation assays demonstrate that ALKBH5 knockdown inhibits cisplatin-sensitive EOC cell proliferation. (E) The transfection effieciency of specific siRNAs targeting ALKBH5 in cisplatin-resistant EOC cells. (F and G) CCK8 and EdU proliferation assays demonstrate that ALKBH5 knockdown also inhibits cisplatin-resistant EOC cell proliferation. (H) ALKBH5 knockdown increases cell sensitivity to cisplatin. (I) γH2AX foci increases after ALKBH5 knockdown in cisplatin-resistant EOC cell.

**Additional file 12 Supplementary Fig. 2** Functional analysis of RNA-seq data. GO analysis based on RNA-seq data showed that DNA repair is enriched in cells with ALKBH5 overexpression

**Additional file 13 Supplementary Fig. 3** HOXA10 downregulation inhibits cell resistance to cisplatin. (A) Correlation analyses in 57 surgical EOC samples (top) and 426 EOC samples in TCGA database (bottom) confirms that HOXA10 expression is positively correlated with ALKBH5 expression in EOC. (B and C) CCK8 and EdU assays demonstrate that HOXA10 knockdown inhibits cisplatin-sensitive EOC cell proliferation. (D-F) CCK8 and EdU assays demonstrate that HOXA10 knockdown inhibits cisplatin-resistant EOC cell proliferation. (G) HOXA10 knockdown increases EOC cell sensitivity to cisplatin. (H) γH2AX foci significantly increases after HOXA10 knockdown in cisplatin-resistant EOC cell.

**Additional file 14 Supplementary Fig. 4** ALKBH5 “erases” m6A modification in EOC cells and correlates with JAK2 expression. (A) m6A dot-blot assay shows that ALKBH5 overexpression significantly decreases the m6A modification level in EOC cells. (B) Correlation analyses of ALKBH5 and JAK2 expression in 57 EOC samples (left) and 426 EOC samples in TCGA database (right). (C) Correlation analyses of HOXA10 and JAK2 expression in 57 EOC samples (left) and 426 EOC samples in TCGA database (right).

**Additional file 15 Supplementary Fig. 5** JAK2 kncockdown suppresses cell proliferation in EOC cells with ALKBH5 and HOXA10 overexpression. (A) The transfection effieciency of specific siRNAs targeting JAK2 in cisplatin-sensitive EOC cells. (B and C) CCK8 proliferation assay shows that JAK2 knockdown inhibits cell proliferation induced by ALKBH5 and HOXA10 overexpression. (D and E) EdU proliferation assay shows that JAK2 knockdown inhibits cell proliferation induced by ALKBH5 and HOXA10 overexpression.

**Additional file 16 Supplementary Fig. 6** JAK2 kncockdown suppresses cell resistance to cisplatin in EOC cells with ALKBH5 and HOXA10 overexpression. (A and B) JAK2 knockdown inhibits cell resistance to cisplatin induced by ALKBH5 and HOXA10 overexpression. (C and D) γH2AX foci significantly increases after JAK2 knockdown in EOC cells with ALKBH5 and HOXA10 overexpression.

**Additional file 17 Supplementary Fig. 7** Inhibition of the JAK2/STAT3 signaling pathway suppresses cisplatin resistance in EOC cells with ALKBH5 and HOXA10 overexpression. (A and B) WP1066 effectively suppresses cancer cell proliferation in EOC cells with ALKBH5 and HOXA10 overexpression. (C and D) WP1066 effectively suppresses cancer cell resistance to cisplatin and DDR in EOC cells with ALKBH5 and HOXA10 overexpression.

## Data Availability

The datasets used and analyzed during the current study are available from the corresponding author on reasonable request.

## Abbreviations

3′-UTR: Three prime untranslated region
Act-D: Actinomycin-D
AGE: Agarose gel electrophoresis
ALKBH5: AlkB homolog 5
CCK8: Cell Counting Kit-8
ChIP: Chromatin immunoprecipitation
CR: Clinical remission
DDR: DNA damage repairment
EOC: Epithelial ovarian cancer
FTO: Fat mass and obesity associated
HOXA10: Homeobox A10
IF: (HOXA10)Immunofluorescence
IHC: Immunohistochemistry
IGF2BP1–3: Insulin-like growth factor-2 mRNA binding proteins
METTL3: N6-methyladenosinemethyltransferase-like m6A 3
METTL14: Methyltransferase-like14
MeRIP-seq: m6A-modified RNA immunoprecipitation sequencing
mRNA: Messenger RNA
Mut: Mutated-type
NCCN: National Comprehensive Cancer Network
NS: Normal saline
PBS: Phosphate buffer saline
RIP: RNA immunoprecipitation
RNA-seq: RNA sequencing
TF: Transcription factor
qPCR: Quantitative Real-time PCR
WT: Wide-type
WTAP: WT1 associated protein
YTHDF: YTH domain family
