# Supplementary material for: ALKBH5-HOXA10 loop-mediated JAK2 m6A demethylation and cisplatin resistance in epithelial ovarian cancer
Source: J Exp Clin Cancer Res. 2021 Sep 8;40:284. doi: 10.1186/s13046-021-02088-1 (PMC8425158; doi:10.1186/s13046-021-02088-1)
Supplement: Supplementary file 4 — Additional file 4. [file 13046_2021_2088_MOESM4_ESM.docx]

| Patient | Age | Stage  (FIGO) | Grade | Histology | Chemotherapy | CR/PR | PFS1  (mouth) |
| --- | --- | --- | --- | --- | --- | --- | --- |
| R001 | 52 | IIC | G3 | Serous adenocarcinoma | [taxol](javascript:;)+paraplatin | Yes | 5 |
| R002 | 60 | IIIC | G3 | Poorly differentiated adenocarcinoma | [taxol](javascript:;)+paraplatin | No | _ |
| R003 | 53 | IIIC | G3 | Serous adenocarcinoma | [taxol](javascript:;)+paraplatin | Yes | 5 |
| R004 | 55 | IIIC | G3 | Serous adenocarcinoma | [taxol](javascript:;)+paraplatin | Yes | 3 |
| R005 | 42 | IIIC | G3 | Serous adenocarcinoma | [taxol](javascript:;)+paraplatin | Yes | 5 |
| R006 | 45 | IIIC | G3 | Serous adenocarcinoma | [taxol](javascript:;)+paraplatin | No | _ |
| R007 | 55 | IIIC | G3 | Serous adenocarcinoma | [taxol](javascript:;)+paraplatin+befazumab | Yes | 5 |
| R008 | 42 | IIIC | G3 | Serous adenocarcinoma | [taxol](javascript:;)+paraplatin+befazumab | Yes | 4 |
| R009 | 37 | IIIC | G3 | Poorly differentiated adenocarcinoma | [taxol](javascript:;)+paraplatin | Yes | 5 |
| S001 | 68 | Ⅳ | G3 | Serous adenocarcinoma | [taxol](javascript:;)+paraplatin | Yes | 14 |
| S002 | 56 | IIIC | G3 | Serous adenocarcinoma | [taxol](javascript:;)+paraplatin | Yes | 20 |
| S003 | 61 | IIIC | G3 | Poorly differentiated adenocarcinoma | docetaxel +paraplatin | Yes | 24 |
| S004 | 50 | IIIC | G3 | Serous adenocarcinoma | [taxol](javascript:;)+paraplatin | Yes | ＞36 |
| S005 | 44 | IIIC | G3 | Serous adenocarcinoma | docetaxel +paraplatin | Yes | ＞36 |
| S006 | 45 | IIIC | G3 | Serous adenocarcinoma | [taxol](javascript:;)+paraplatin | Yes | 18 |
| S007 | 33 | IIC | G3 | Serous adenocarcinoma | [taxol](javascript:;)+paraplatin | Yes | ＞36 |
| S008 | 64 | IIIC | G3 | Poorly differentiated adenocarcinoma | [taxol](javascript:;)+paraplatin | Yes | 21 |
| S009 | 53 | IIIC | G3 | Poorly differentiated adenocarcinoma | [taxol](javascript:;)+paraplatin | Yes | 24 |
| S010 | 47 | IIIC | G3 | Serous adenocarcinoma | docetaxel +paraplatin | Yes | 18 |
| S011 | 40 | IIIC | G3 | Poorly differentiated adenocarcinoma | docetaxel +paraplatin | Yes | ＞36 |
| S012 | 54 | IIIC | G3 | Poorly differentiated adenocarcinoma | [taxol](javascript:;)+paraplatin | Yes | 15 |
| S013 | 46 | IIIC | G3 | Poorly differentiated adenocarcinoma | [taxol](javascript:;)+paraplatin | Yes | 28 |
| S014 | 50 | IIIC | G3 | Serous adenocarcinoma | [taxol](javascript:;)+paraplatin+ befazumab | Yes | ＞36 |
| S015 | 50 | IIIC | G3 | Poorly differentiated adenocarcinoma | [taxol](javascript:;)+paraplatin | Yes | ＞36 |

**Supplementary Table 1: The descriptive information of 15 sensitive- and 9 resistant- EOC patients.**
