# Supplementary material for: ALKBH5-HOXA10 loop-mediated JAK2 m6A demethylation and cisplatin resistance in epithelial ovarian cancer
Source: J Exp Clin Cancer Res. 2021 Sep 8;40:284. doi: 10.1186/s13046-021-02088-1 (PMC8425158; doi:10.1186/s13046-021-02088-1)
Supplement: Supplementary file 5 — Additional file 5. [file 13046_2021_2088_MOESM5_ESM.docx]

| Gene | Forward (5´-3´) | Reverse (5´-3´) |
| --- | --- | --- |
| ALKBH5 | CGGCGAAGGCTACACTTACG | CCACCAGCTTTTGGATCACCA |
| FTO | AACACCAGGCTCTTTACGGTC | TGTCCGTTGTAGGATGAACCC |
| GAPDH | GCACCGTCAAGGCTGAGAAC | GGATCTCGCTCCTGGAAGATG |
| HOXA10 | CTCGCCCATAGACCTGTGG | GTTCTGCGCGAAAGAGCAC |
| JAK2 | TCTGGGGAGTATGTTGCAGAA | AGACATGGTTGGGTGGATACC |
| METTL3 | TTGTCTCCAACCTTCCGTAGT | CCAGATCAGAGAGGTGGTGTAG |
| METTL14 | GAGTGTGTTTACGAAAATGGGGT | CCGTCTGTGCTACGCTTCA |
| WTAP | CTTCCCAAGAAGGTTCGATTGA | TCAGACTCTCTTAGGCCAGTTAC |
| YTHDF2 | AGCCCCACTTCCTACCAGATG | TGAGAACTGTTATTTCCCCATGC |
| Specific primer of JAK2 for MeRIP-qPCR | TGTAAATCAACGCCCCTCCT | GGTCATTTCTTTCATCCAGCCA |
| Specific primer of ALKBH5 for ChIP-qPCR | CAAAGGGGGAGGTAACGGAG | GGTGCGCTCATATTGCCAAC |

**Supplementary Table 2：Primers used for qPCR assay.**
