# Supplementary material for: ALKBH5-HOXA10 loop-mediated JAK2 m6A demethylation and cisplatin resistance in epithelial ovarian cancer
Source: J Exp Clin Cancer Res. 2021 Sep 8;40:284. doi: 10.1186/s13046-021-02088-1 (PMC8425158; doi:10.1186/s13046-021-02088-1)
Supplement: Supplementary file 6 — Additional file 6. [file 13046_2021_2088_MOESM6_ESM.docx]

**Supplementary Table 3：Antibodies used in this study.**

| Antibodies | Antibodies |
| --- | --- |
| Anti-ALKBH5 | ab244296, Abcam |
| Anti-HOXA10 | ab191470, Abcam |
| Anti-JAK2 | 17670-1-AP, Proteintech |
| Anti-p-STAT3 | ab76315, Abcam |
| Anti-YTHDF2 | 24744-1-AP, Proteintech Group |
| Anti-Flag | ab205606, Abcam |
| Anti-GAPDH | ab59164, Abcam |
| Anti-γH2AX | ab81299, Abcam |
| Alexa Fluor 594 goat Anti-rabbit IgG | ab150080, Abcam |
| anti-rabbit antibody | ab206718, Abcam |
| Anti-m6A | ab232905, Abcam |
