# Supplementary material for: ALKBH5-HOXA10 loop-mediated JAK2 m6A demethylation and cisplatin resistance in epithelial ovarian cancer
Source: J Exp Clin Cancer Res. 2021 Sep 8;40:284. doi: 10.1186/s13046-021-02088-1 (PMC8425158; doi:10.1186/s13046-021-02088-1)
Supplement: Supplementary file 7 — Additional file 7. [file 13046_2021_2088_MOESM7_ESM.docx]

**Supplementary Table 4. RNAi oligonucleotides sequences.**

| siRNA | RNAi oligonucleotides sequences (5´-3´) |
| --- | --- |
| siALKBH5-1 | UCAGAUCGCCUGUCAGGAATT |
| siALKBH5-2 | GGA UAU GCU GCU GAU GAA ATT |
| siHOXA10-1 | GCAAAGAGUGGUCGGAAGATT |
| siHOXA10-2 | CACGGACAGACAAGUCAAATTUUUCACUUGUCUGUCCGUGTT |
| siYTHDF2-1 | TTGGCTATGGGAACGTCTT |
| siYTHDF2-2 | CAAGGAAACAAAGTGCAAA |
| siJAK2-1 | GCAAAUAGAUCCAGUCCUATT |
| siJAK2-2 | CAAGAGGGUUCAAAUGAAATT |
