# Supplementary material for: ALKBH5-HOXA10 loop-mediated JAK2 m6A demethylation and cisplatin resistance in epithelial ovarian cancer
Source: J Exp Clin Cancer Res. 2021 Sep 8;40:284. doi: 10.1186/s13046-021-02088-1 (PMC8425158; doi:10.1186/s13046-021-02088-1)
Supplement: Supplementary file 8 — Additional file 8. [file 13046_2021_2088_MOESM8_ESM.docx]

**Supplementary Table 5**：**Detailed DNA sequences of luciferase reporter**

| Luciferase reporter | Position | DNA sequences |
| --- | --- | --- |
| JAK2 WT | >NC_000009.12:5126686-5127015 Homo sapiens chromosome 9, GRCh38.p13 Primary Assembly | CUAUAUGAUCAUGACAGAAUGCUGGAACAAUAAUGUAAAUCAACGCCCCUCCUUUAGGGAUCUAGCUCUU  CGAGUGGAUCAAAUAAGGGAUAACAUGGCUGGAUGAAAGAAAUGACCUUCAUUCUGAGACCAAAGUAGAU  UUACAGAACAAAGUUUUAUAUUUCACAUUGCUGUGGACUAUUAUUACAUAUAUCAUUAUUAUAUAAAUCA  UGAUGCUAGCCAGCAAAGAUGUGAAAAUAUCUGCUCAAAACUUUCAAAGUUUAGUAAGUUUUUCUUCAUG  AGGCCACCAGUAAAAGACAUUAAUGAGAAUUCCUUAGCAAGGAUUUUGUA |
| JAK2 Mut | >NC_000009.12:5126686-5127015 Homo sapiens chromosome 9, GRCh38.p13 Primary Assembly | CUAUAUGAUCAUGACAGAAUGCUGGACCAAUAAUGUAAAUCAACGCCCCUCCUUUAGGGAUCUAGCUCUU  CGAGUGGAUCAAAUAAGGGAUAACAUGGCUGGAUGAAAGAAAUGACCUUCAUUCUGAGACCAAAGUAGAU  UUACAGACCAAAGUUUUAUAUUUCACAUUGCUGUGGCCUAUUAUUACAUAUAUCAUUAUUAUAUAAAUCA  UGAUGCUAGCCAGCAAAGAUGUGAAAAUAUCUGCUCAAAACUUUCAAAGUUUAGUAAGUUUUUCUUCAUG  AGGCCACCAGUAAAAGACAUUAAUGAGAAUUCCUUAGCAAGGAUUUUGUA |
| ALKBH5 WT | >NC_000017.11:18181828-18184028 Homo sapiens chromosome 17, GRCh38.p13 Primary Assembly | CCCTATATATACAATGGTAATAATAATAATGCCCTCCTCATTGAATTGTTTGGACGATTAATTGGGCTAG  TATATGCACAGGTCCTGGCACATAGTAAAAACTTGATAAGCTTTTTAAAAAAATGTCCATTCCAGGCTGG |
| ALKBH5 Mut | >NC_000017.11:18181828-18184028 Homo sapiens chromosome 17, GRCh38.p13 Primary Assembly | CCCTATATATACAATGGGGCGCCGGGGGGTGCCCTCCTCATTGAATTGTTTGGACGCGGCGCCGGGCTAG  TATATGCACAGGTCCTGGCACCGGGGCCGGGCTTGATAAGCTTCCGGCGCCGGGGTCCATTCCAGGCTGG |
