# Supplementary material for: ALKBH5-HOXA10 loop-mediated JAK2 m6A demethylation and cisplatin resistance in epithelial ovarian cancer
Source: J Exp Clin Cancer Res. 2021 Sep 8;40:284. doi: 10.1186/s13046-021-02088-1 (PMC8425158; doi:10.1186/s13046-021-02088-1)
Supplement: Supplementary file 10 — Additional file 10. [file 13046_2021_2088_MOESM10_ESM.docx]

| Characteristic | | All | | HOXA10 expression | | *p*-value |
| --- | --- | --- | --- | --- | --- | --- |
|  |  |  |  | Low-expression | High-expression |  |
| Total | | 57 | | | | |
| Age at surgery | | | | | | 0.772 |
| ≥60 | 13 | | | 8 | 5 | |
| ＜60 | 44 | | | 29 | 15 | |
| Histology | | | | | | 0.272 |
| Serous | | 35 | | 20 | 15 | |
| Mucinous | | 11 | | 8 | 3 | |
| Other | | 11 | | 9 | 2 | |
| Grade | | | | | | 0.273 |
| G1/G2 | | 16 | | 10 | 6 | |
| G3 | | 41 | | 19 | 22 | |
| Stage | | | | | | 0.07 |
| I/II | | 20 | | 17 | 3 | |
| III/IV | | 37 | | 20 | 17 | |
| Tumor size | | | | | | 0.004^**^ |
| ≥8cm | | | 28 | 13 | 15 | |
| ＜8cm | | | 29 | 24 | 5 | |
| Lymph node | |  | |  |  | 0.011^*^ |
| Positive | | 19 | | 8 | 11 | |
| Negative | | 38 | | 29 | 9 | |
| Peritoneal cytology | | | | | | 0.704 |
| Positive | | 41 | | 21 | 15 | |
| Negative | | 16 | | 11 | 5 | |

**Supplementary Table 7**

**The descriptive analysis of HOXA10 mRNA expression and patients’ clinical characteristics**
