# Supplementary figures and images for: ALKBH5-HOXA10 loop-mediated JAK2 m6A demethylation and cisplatin resistance in epithelial ovarian cancer
Source: J Exp Clin Cancer Res. 2021 Sep 8;40:284. doi: 10.1186/s13046-021-02088-1 (PMC8425158; doi:10.1186/s13046-021-02088-1)

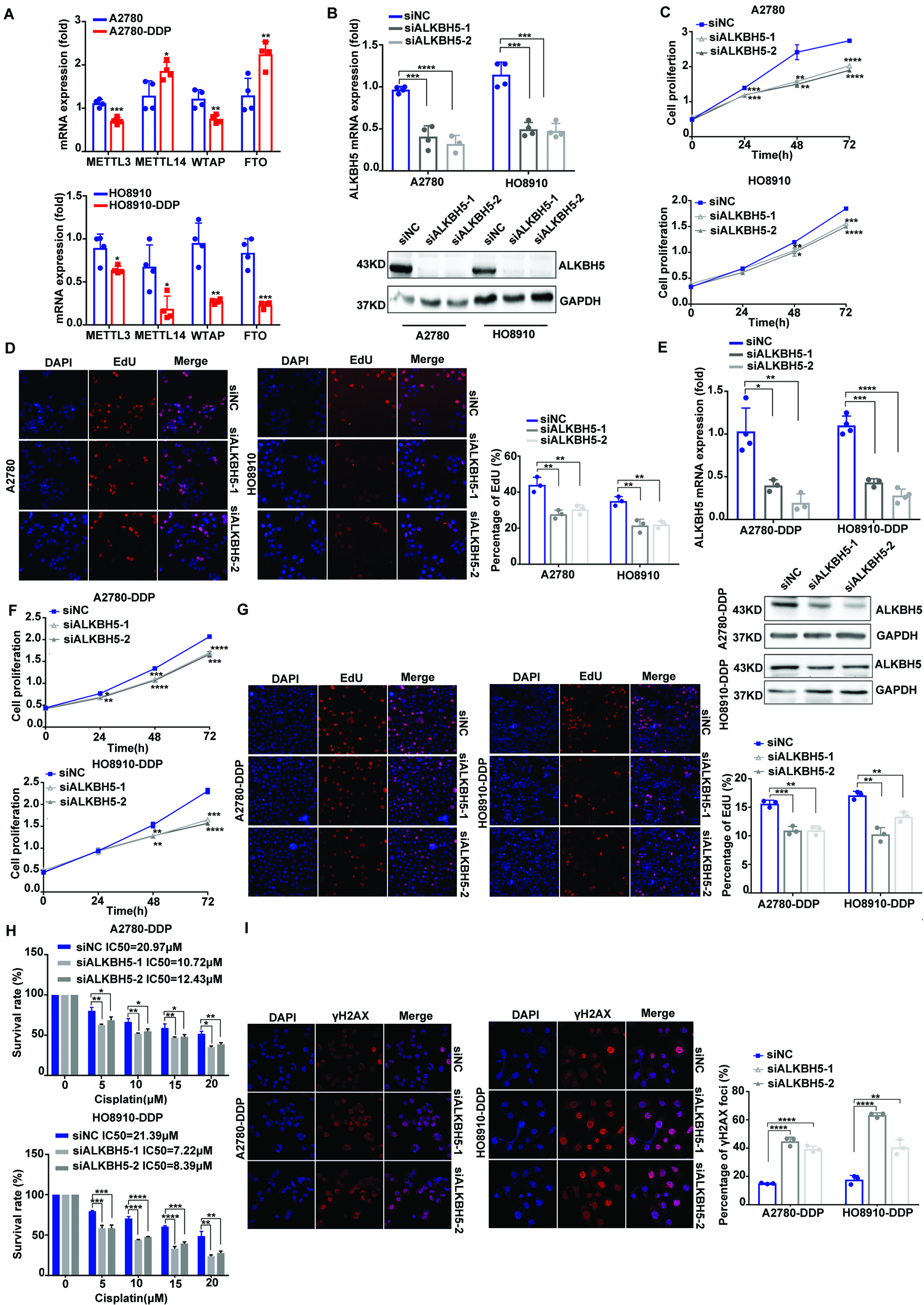

Supplement: Supplementary file 11 — Additional file 11 Supplementary Fig. 1 ALKBH5 downregulation inhibits cell resistance to cisplatin. (A) The mRNA expression of METTL3, METTL14, WTAP and FTO is validiated in cisplatin-sensitive and cisplatin-resistant EOC cells. (B) The transfection effieciency of specific siRNAs targeting ALKBH5 in cisplatin-sensitive EOC cells. (C and D) CCK8 and EdU proliferation assays demonstrate that ALKBH5 knockdown inhibits cisplatin-sensitive EOC cell proliferation. (E) The transfection effieciency of specific siRNAs targeting ALKBH5 in cisplatin-resistant EOC cells. (F and G) CCK8 and EdU proliferation assays demonstrate that ALKBH5 knockdown also inhibits cisplatin-resistant EOC cell proliferation. (H) ALKBH5 knockdown increases cell sensitivity to cisplatin. (I) γH2AX foci increases after ALKBH5 knockdown in cisplatin-resistant EOC cell. [file 13046_2021_2088_MOESM11_ESM.tif]

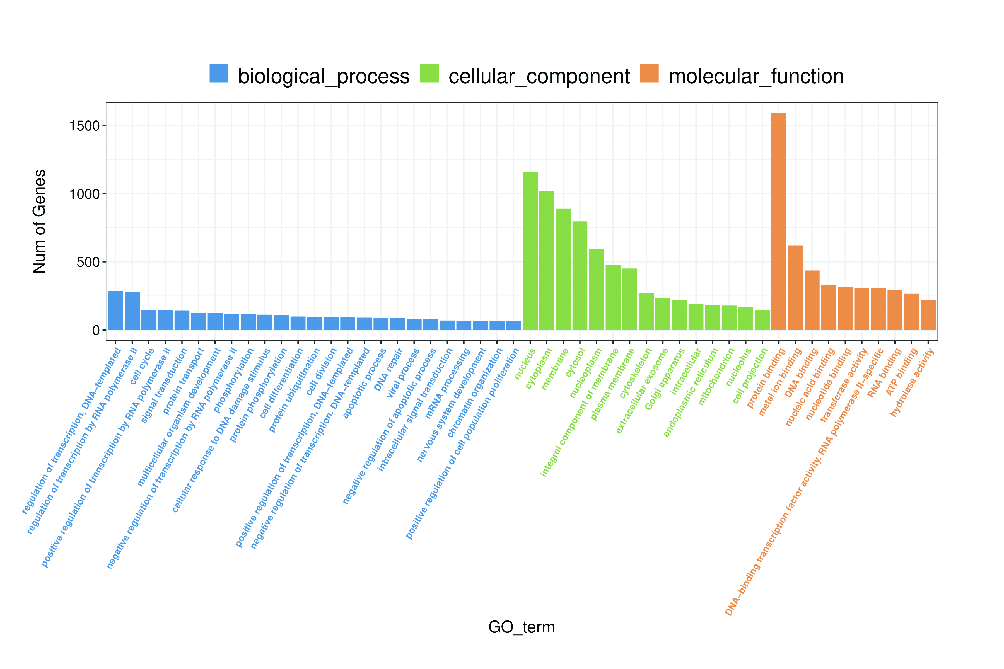

Supplement: Supplementary file 12 — Additional file 12 Supplementary Fig. 2 Functional analysis of RNA-seq data. GO analysis based on RNA-seq data showed that DNA repair is enriched in cells with ALKBH5 overexpression [file 13046_2021_2088_MOESM12_ESM.tif]

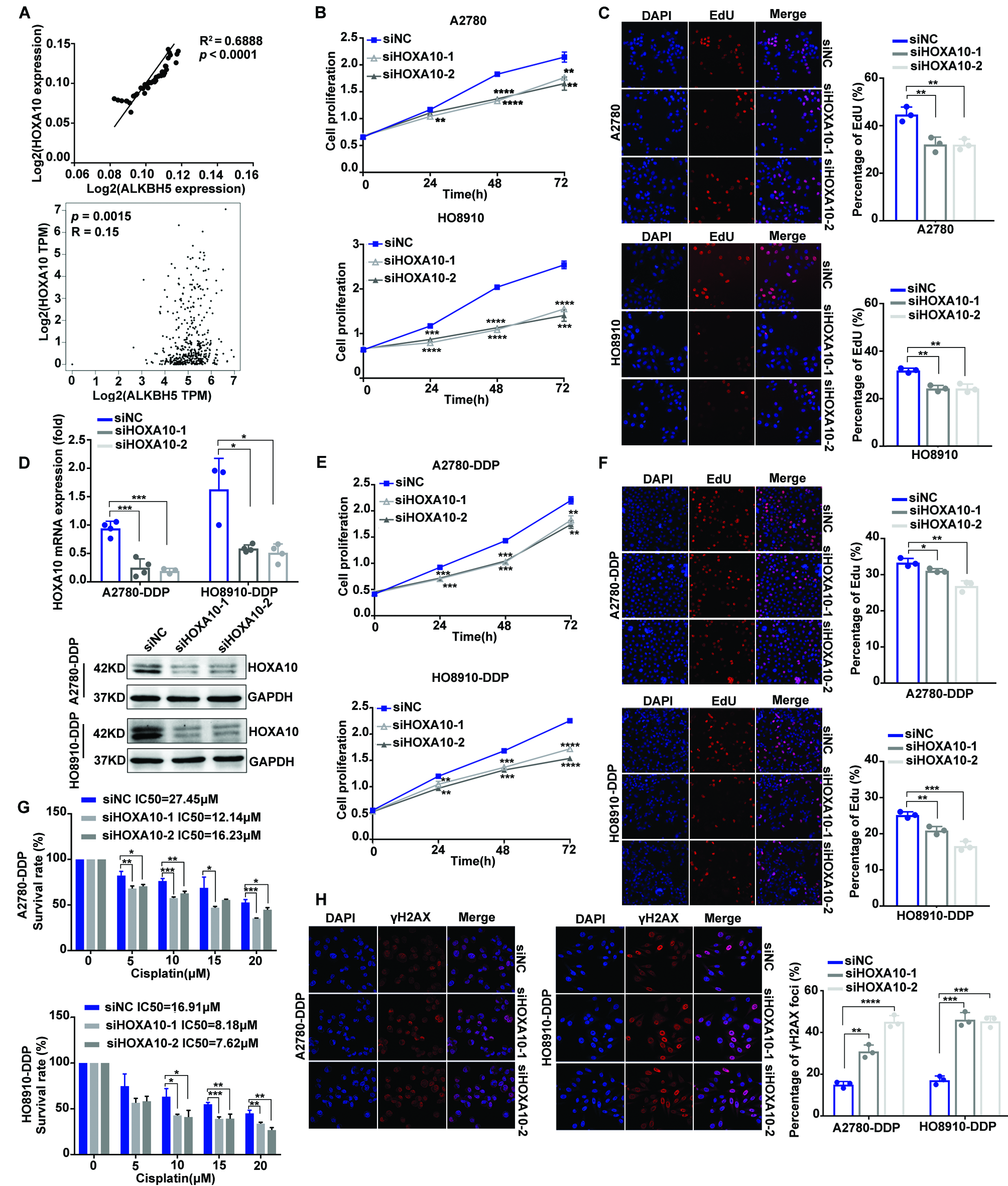

Supplement: Supplementary file 13 — Additional file 13 Supplementary Fig. 3 HOXA10 downregulation inhibits cell resistance to cisplatin. (A) Correlation analyses in 57 surgical EOC samples (top) and 426 EOC samples in TCGA database (bottom) confirms that HOXA10 expression is positively correlated with ALKBH5 expression in EOC. (B and C) CCK8 and EdU assays demonstrate that HOXA10 knockdown inhibits cisplatin-sensitive EOC cell proliferation. (D-F) CCK8 and EdU assays demonstrate that HOXA10 knockdown inhibits cisplatin-resistant EOC cell proliferation. (G) HOXA10 knockdown increases EOC cell sensitivity to cisplatin. (H) γH2AX foci significantly increases after HOXA10 knockdown in cisplatin-resistant EOC cell. [file 13046_2021_2088_MOESM13_ESM.tif]

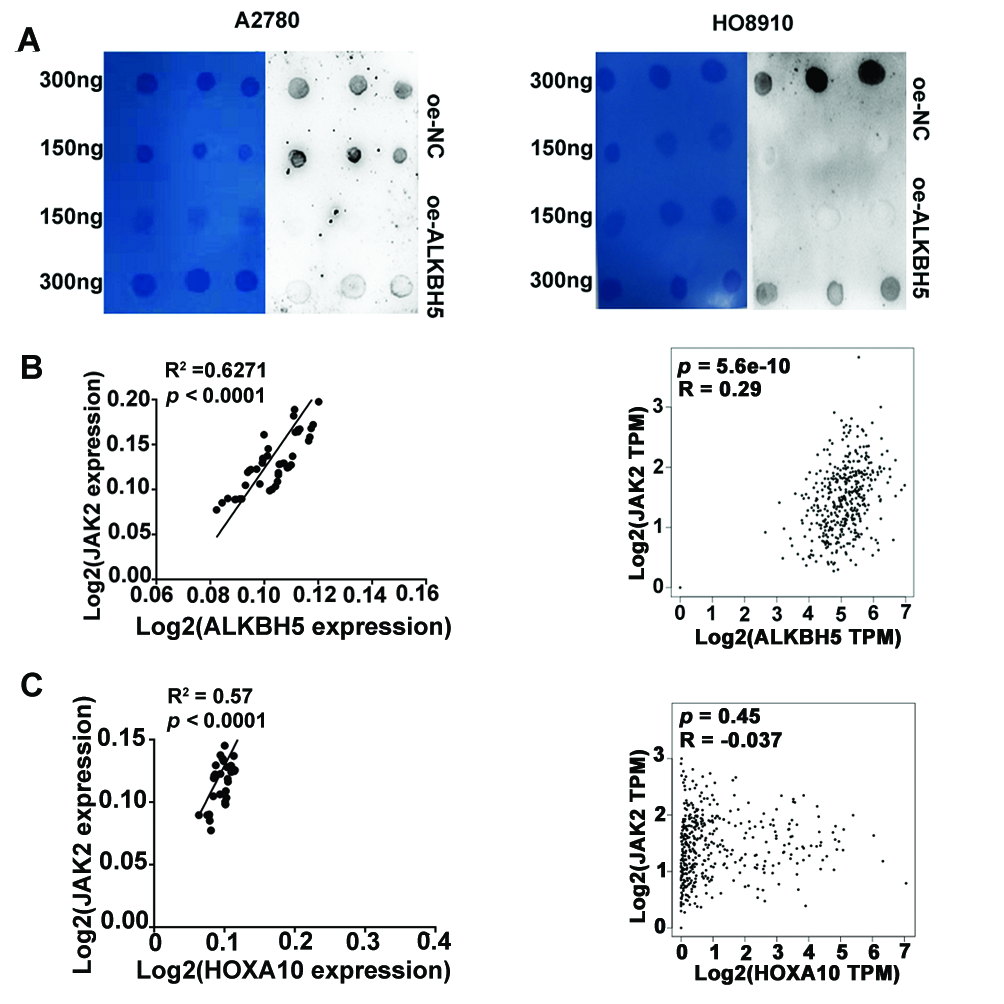

Supplement: Supplementary file 14 — Additional file 14 Supplementary Fig. 4 ALKBH5 “erases” m6A modification in EOC cells and correlates with JAK2 expression. (A) m6A dot-blot assay shows that ALKBH5 overexpression significantly decreases the m6A modification level in EOC cells. (B) Correlation analyses of ALKBH5 and JAK2 expression in 57 EOC samples (left) and 426 EOC samples in TCGA database (right). (C) Correlation analyses of HOXA10 and JAK2 expression in 57 EOC samples (left) and 426 EOC samples in TCGA database (right). [file 13046_2021_2088_MOESM14_ESM.tif]

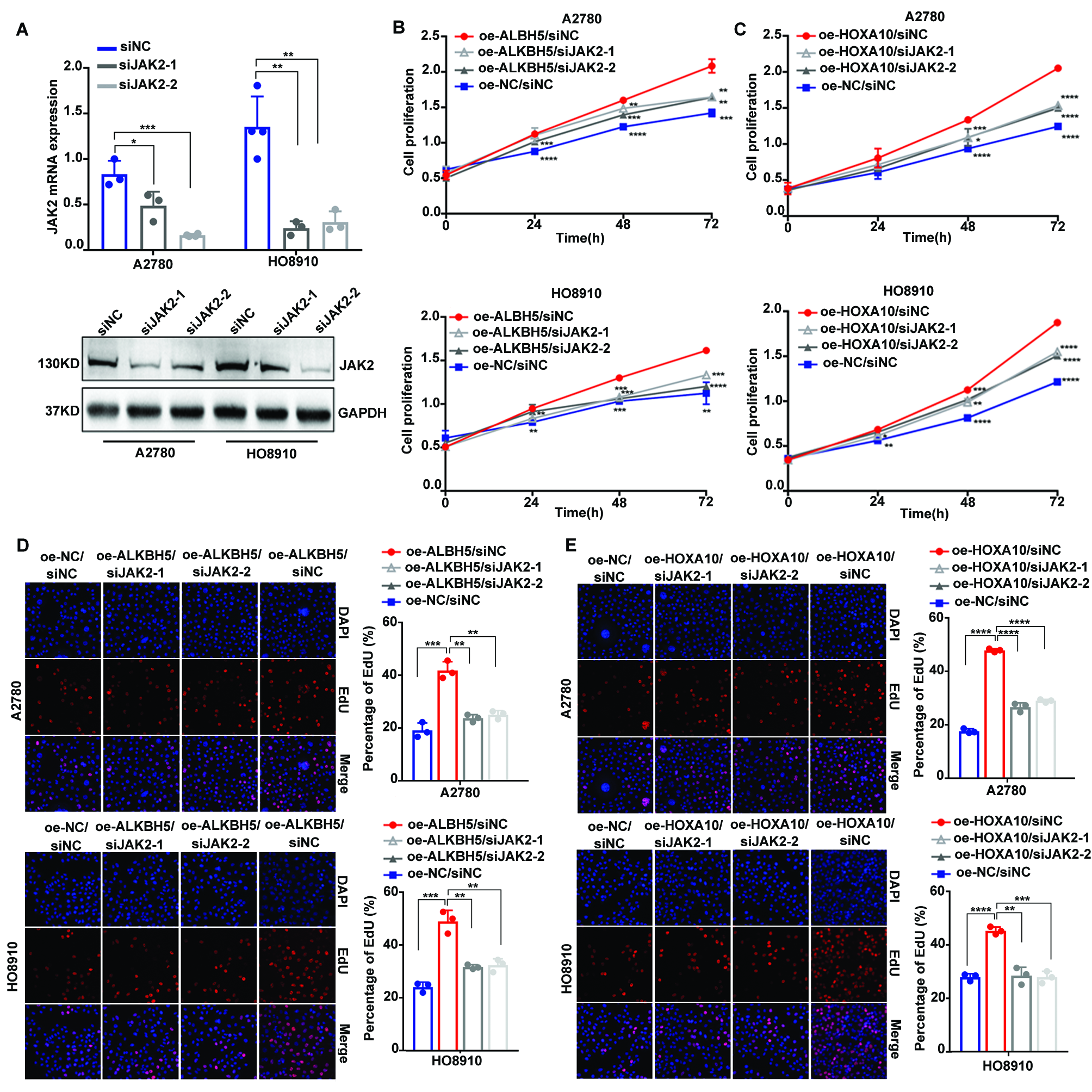

Supplement: Supplementary file 15 — Additional file 15 Supplementary Fig. 5 JAK2 kncockdown suppresses cell proliferation in EOC cells with ALKBH5 and HOXA10 overexpression. (A) The transfection effieciency of specific siRNAs targeting JAK2 in cisplatin-sensitive EOC cells. (B and C) CCK8 proliferation assay shows that JAK2 knockdown inhibits cell proliferation induced by ALKBH5 and HOXA10 overexpression. (D and E) EdU proliferation assay shows that JAK2 knockdown inhibits cell proliferation induced by ALKBH5 and HOXA10 overexpression. [file 13046_2021_2088_MOESM15_ESM.tif]

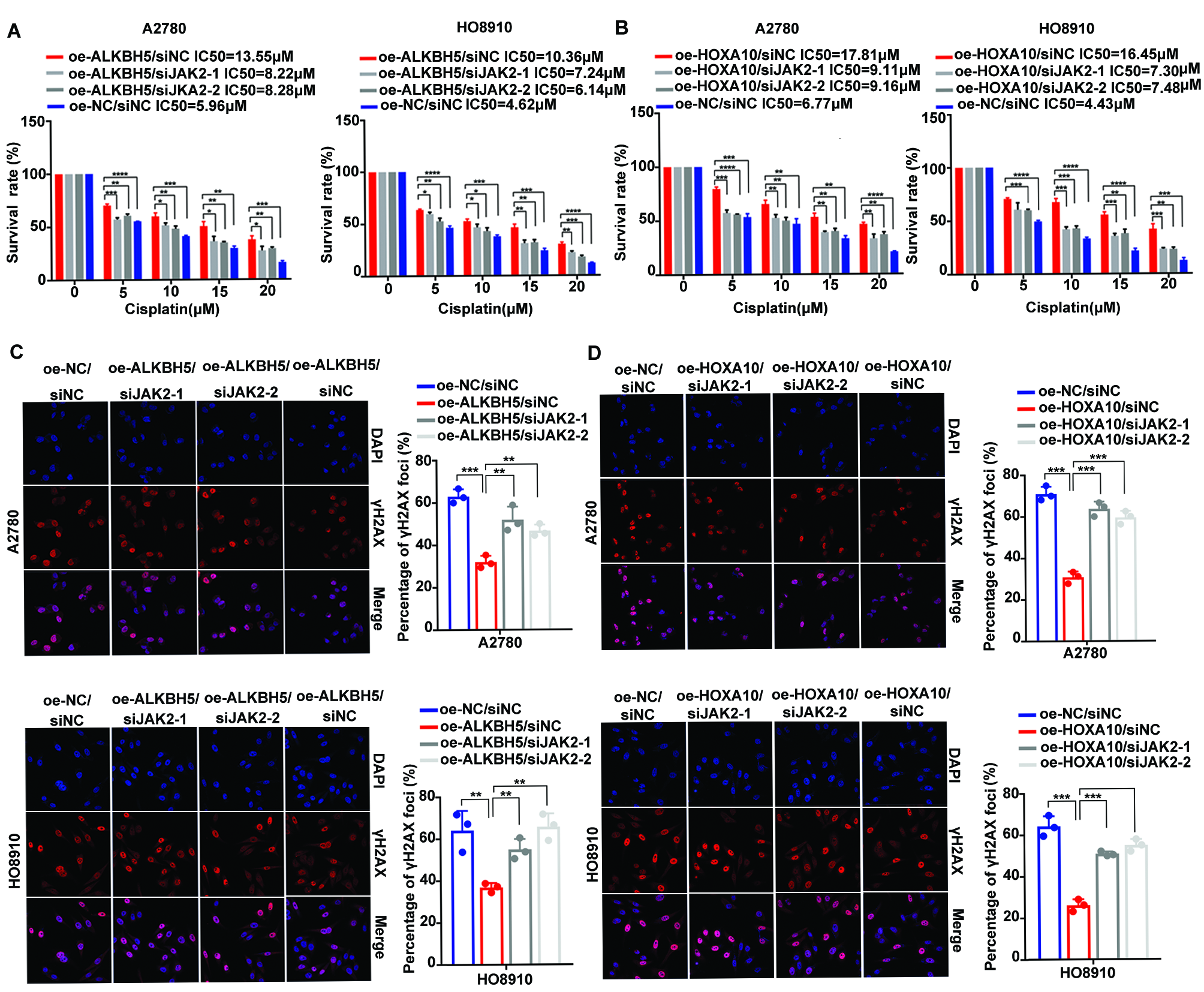

Supplement: Supplementary file 16 — Additional file 16 Supplementary Fig. 6 JAK2 kncockdown suppresses cell resistance to cisplatin in EOC cells with ALKBH5 and HOXA10 overexpression. (A and B) JAK2 knockdown inhibits cell resistance to cisplatin induced by ALKBH5 and HOXA10 overexpression. (C and D) γH2AX foci significantly increases after JAK2 knockdown in EOC cells with ALKBH5 and HOXA10 overexpression. [file 13046_2021_2088_MOESM16_ESM.tif]

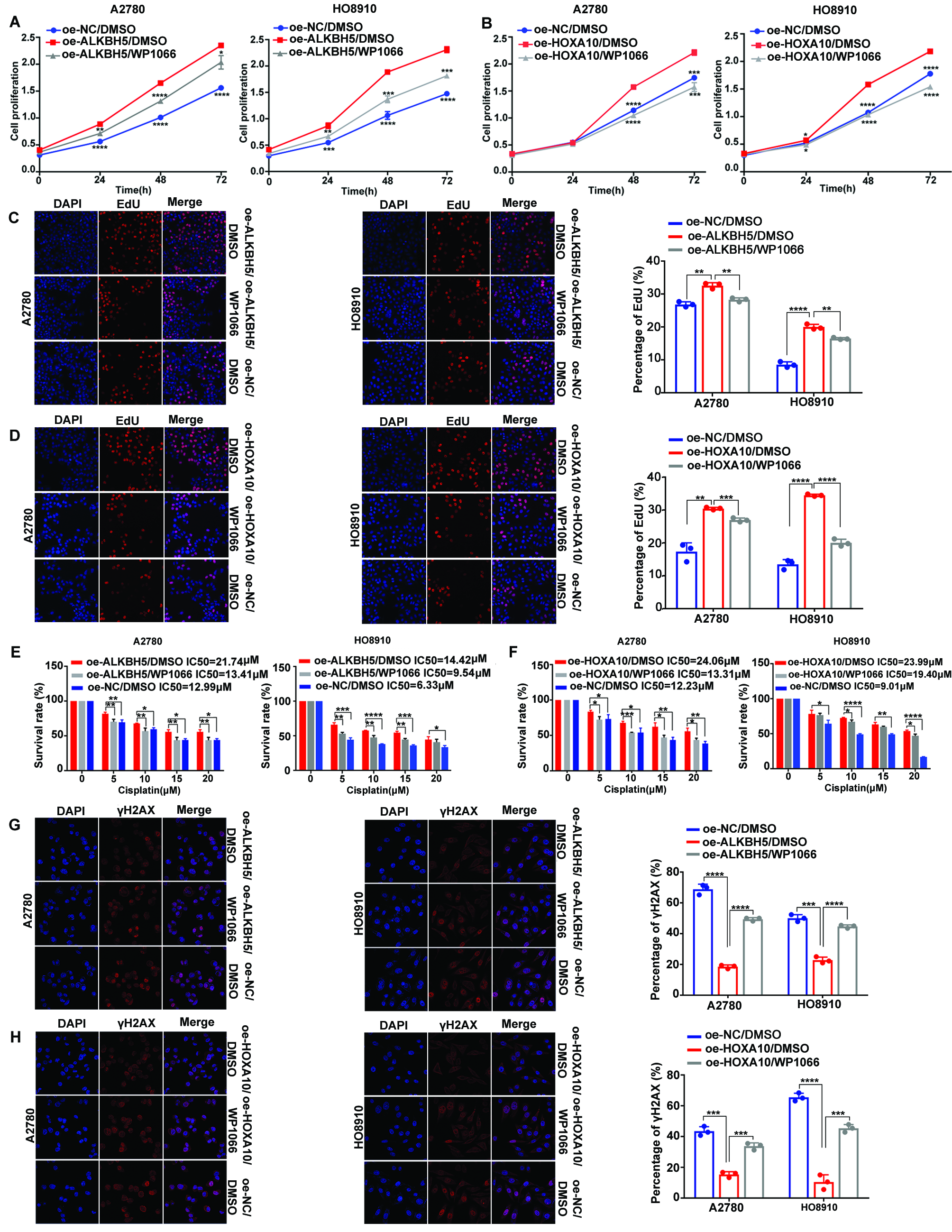

Supplement: Supplementary file 17 — Additional file 17 Supplementary Fig. 7 Inhibition of the JAK2/STAT3 signaling pathway suppresses cisplatin resistance in EOC cells with ALKBH5 and HOXA10 overexpression. (A and B) WP1066 effectively suppresses cancer cell proliferation in EOC cells with ALKBH5 and HOXA10 overexpression. (C and D) WP1066 effectively suppresses cancer cell resistance to cisplatin and DDR in EOC cells with ALKBH5 and HOXA10 overexpression. [file 13046_2021_2088_MOESM17_ESM.tif]
